# The genetic link between thyroid dysfunction and alopecia areata: a bidirectional two-sample Mendelian randomization study

**DOI:** 10.3389/fendo.2024.1440941

**Published:** 2024-08-14

**Authors:** Le Gao, Wenrui Li, Qiang Song, Hengxing Gao, Mingwei Chen

**Affiliations:** ^1^ Department of Respiratory and Critical Care Medicine, First Affiliated Hospital of Xi’an Jiaotong University, Xi’an, China; ^2^ Department of Structural Heart Disease, First Affiliated Hospital of Xi’an Jiaotong University, Xi’an, China

**Keywords:** alopecia areata, causal relationship, Hashimoto’s thyroiditis, thyroid dysfunction, two-sample Mendelian randomization

## Abstract

**Background:**

Although descriptive studies have found an association between thyroid dysfunction (TD) and alopecia areata (AA), however, the causal relationship between TD and AA remains unclear. The purpose of this study is to investigate the causal relationship between the two and the specific directions.

**Methods:**

We performed large-scale, two-sample Mendelian randomization (MR) analyses to examine whether there was an association between TD (such as Graves’ disease (GD), Hashimoto’s thyroiditis (HT), thyroid cancer (TC), thyroid stimulating hormone (TSH), thyrotropin-releasing hormone (TRH), etc.) and AA. Genome-wide association study (GWAS) summary statistics for TD and AA were from the IEU OpenGwas project. The inverse variance-weighted (IVW) method was used as the primary analysis method to evaluate the causality between TD and AA, supplemented by the weighted median, MR-Egger, simple mode and weighted mode. In addition, sensitivity analyses were performed to assess the reliability of the study results.

**Results:**

Our study found that single nucleotide polymorphisms (SNPs) in HT (IVW OR = 1.396, 95% CI 1.030-1.892, *P*=0.031) and hypothyroidism (IVW OR = 1.431, 95% CI 1.138-1.799, *P*=0.002) significantly increased the risk of AA. Reverse MR analysis indicated that genetic susceptibility to AA (β=-0.029, 95%CI=-0.051 to -0.007, *P*=0.009) may be a risk for TRH. Positive MR analysis observed no statistically significant causal relationship between other TD and AA (IVW *P*>0.05). Reverse MR analysis also showed no statistically significant association between AA and other TD (IVW *P*>0.05) other than TRH. Furthermore, additional sensitivity analyses were performed, including a leave-one-out test, a heterogeneity test, and a pleiotropy test to assess the robustness of the results.

**Conclusions:**

This study provides a very comprehensive analysis of the causal relationship between TD and AA, providing convincing genetic evidence to support the causal relationship between TD and alopecia areata. It reveals some causes of AA patients, which is of great significance for the management and treatment of AA patients.

## 1 Introduction

Alopecia areata (AA) is a common autoimmune disease characterized by non-scarring alopecia. The global prevalence of the disease is about 2% (1, 2), and the prevalence in China is 0.27% (3). The symptoms and signs of AA vary depending on the severity of the condition, from patchy hair loss to diffuse hair involvement on the scalp or the whole body (4, 5). Most AA patients experience unpredictable relapses and remissions (6). The disease not only reduces the quality of life of patients, but also may lead to emotional disorders such as depression and anxiety (7, 8).Further associations between AA and certain inflammatory, metabolic and autoimmune diseases have been observed, increasing the probability of developing them (9, 10); however, causality remains to be established and the exact pathogenesis of AA is still to be discovered. Consequently, comprehending the potential pathogenesis of AA is of utmost importance in order to facilitate the development of efficacious therapeutic approaches and enhance prognosis.

Non-scarring alopecia is a complex process involving genetic predisposition, environmental triggers, impaired hair growth, and inflammatory and immune factors, which may lead to its pathological mechanism (11). The exact pathogenesis of AA has not been completely established; however, a study has shown that alopecia areata is a T cell-mediated autoimmune state due to the collapse of immune privilege in hair follicles (12). Among them, the abnormal thyroid hormone (TH) level and antithyroid autoantibodies have been frequently reported in AA patients, and screening tests for thyroid dysfunction are sometimes recommended for patients with AA (13–16). The effects of thyroid hormone on hair growth have been the subject of special research in recent years, and previous studies have provided strong evidence linking TH to hair loss (17). One comparative study found that the hypothyroidism group (34%) had a higher proportion of patients with severe hair loss compared with the normal thyroid function group (18%) and the hyperthyroidism group (20%) (18). Observational studies have found a significant increase in the incidence of thyroid disease in patients with AA (19). A meta-analysis of patients with AA found that the prevalence of GD, hypothyroidism, TD, and HT in AA patients was 1.4%, 2.3%, 13.3%, and 2.9%, respectively (20). Extensive observational studies have revealed the link between AA and thyroid disease. However, the causal relationship between the two is not yet clear. Furthermore, observational studies may mask the real causal relationship due to reverse causality, selection bias, and confounding factors. Consequently, it is crucial to investigate the correlation between AA and TD in order to gain insights into the fundamental mechanisms of these diseases and improve their therapeutic approaches and quality of life.

Priority of the double-blind randomized controlled trial (RCT) may be compromised due to inherent disadvantages, including challenging ethical approval, substantial time, human and financial investments (21). Mendelian randomization (MR) analysis, a strategy for investigating causation between different traits, is widely used to explore the casual correlation between an exposure and an outcome (22). By including exposure-associated genetic variants of interest as instrumental variables(IVs), MR can avoid unmeasured confounding factors in observational studies and examine the causal relationship between potentially modifiable risk factors and health outcomes (23). Therefore, a two-sample MR analysis was performed in our study, and SNPs strongly correlated with exposure were collected as IVs to assess the causal relationship between AA and TD. This investigation was based on twelve extensive GWAS summary statistics, predominantly focusing on the European population’s data pertaining to AA and TD, including GD, HT, hyperthyroidism, hypothyroidism, thyroid cancer (TC), TSH, thyrotropin-releasing hormone (TRH), thyroxine-binding globulin (TBG), thyroid hormone receptor alpha (THRα), thyroid peroxidase (TP) and thyroglobulin (TG). In addition, in order to test the reliability of the study results, we performed various sensitivity analyses, including heterogeneity test, pleiotropy test, leave-one-out test, and reverse MR analysis.

## 2 Methods

### 2.1 STROBE-MR (strengthening the reporting of observational studies in epidemiology using mendelian randomisation) checklist

This study was guided by the STROBE-MR guidelines. This article adheres to the STROBE-MR checklist for reporting. (**Supplementary Table S5**).

### 2.2 Source of data and study design

The analysis was conducted using publicly accessible summary-level data from GWAS that specifically examined traits of interest, primarily in individuals of European ancestry, encompassing both males and females. A total of 12 datasets on AA and TD traits were collected. Original data for alopecia areata came from the FinnGen study (accessed through https://www.finngen.fi/en/access_results).The FinnGen study was designed to collect and analyze genomic information from more than 500,000 participants from the Finnish Biobank and combine it with information from national healthcare registries (24). AITD (Autoimmune thyroid disease) dataset includes both HT (n=395640) and GD (n=458620). The GWAS dataset associated with GD and HT was derived from the UK Biobank Project (25). Hyperthyroidism dataset (n= 460499) and Hypothyroidism dataset (n=410141) were also derived from the British Biobank project. Data on genetic variants associated with thyroid cancer were obtained by Deutsches the Krebsforschungszentrum (DKFZ) through GWAS (26) and included 1080 European participants, including 649 in the case group and 431 in the control group. TSH, TRH, THRα, TP and TG dataset all contained 3301 individuals in European population (27). TBG dataset was obtained from GWAS through the human blood plasma proteome (28).

**Figure 1:**
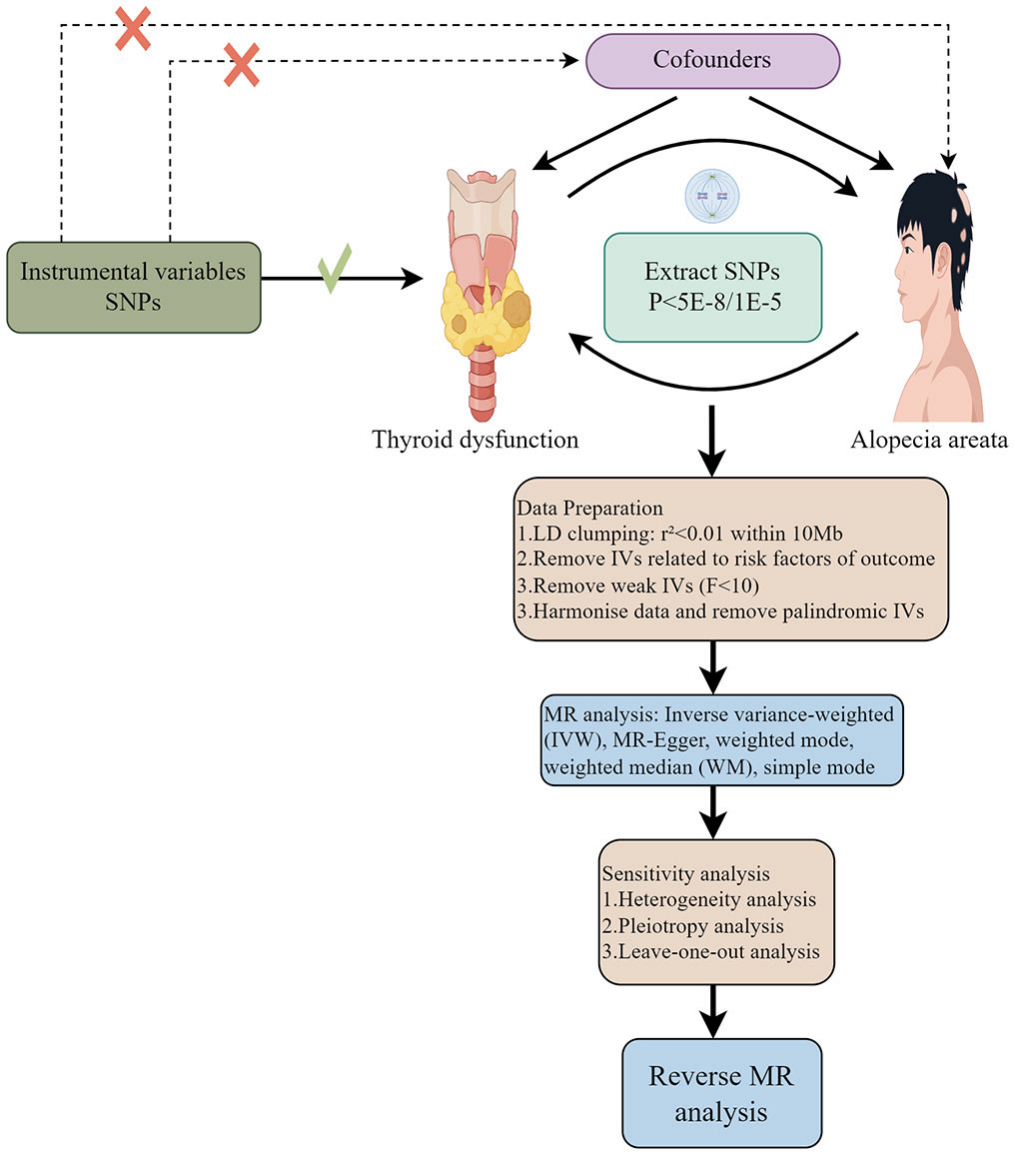
The overall design flow chart of this study.

The flowchart of two-sample MR analyses in this study is shown in **Figure 1**. The exposed and outcome populations were Europeans, avoiding the bias caused by population stratification.

Ethical approval is not sought as the datasets in this study are publicly available.

### 2.3 Variants selection criterion

Single nucleotide polymorphisms (SNPs) were selected as instrumental variables (IVs). In order to make these SNPs show higher statistical power in genetic variation analysis, a series of quality control procedures were implemented.

The legitimate IVs should meet the following assumptions: (1) IVs (genetic variation) are closely related to TD (exposure). (2) genetic variants do not share common causes (potential confounders) with alopecia areata-related indicators (outcomes). (3) genetic variation affects alopecia areata-related indicators (outcomes) only through its effect on thyroid dysfunction-related traits (exposure). In forward MR analysis, *P* value significance threshold of the SNP was set to 5×10^-8^ (29, 30). In the event that the number of filtered SNPs was insufficient, it was feasible to modify the threshold to *P*<1×10^-5^ (31). Next, linkage disequilibrium (LD) pruning was conducted to remove linked SNPs (R^2^<0.001, kb=10000).

No proxy SNP was used in this MR analysis. Nevertheless, it remains possible that some pleiotropic SNPs, which are difficult to detect, might still exist. To further evaluate whether each SNP is associated with established risk factors of AA, including genetic factors (32), mental stress (Such as anxiety, depression, insomnia, etc.) (33) and intestinal dysbiosis (34), we utilized the LDlink website (https://ldlink.nih.gov/?tab=ldtrait) (35) to scrutinize exposure-related instrumental variables (IVs). If we identify any SNPs are significantly linked to the aforementioned confounding factors (*P*< 1 × 10^− 8^), we will exclude these SNPs and re-conduct the MR analysis. This crucial step aims to ensure the robustness and reliability of our analysis results. IVs intensity was measured by calculating the F-statistic (36). SNPs with F-statistics smaller than 10 were removed (30). In reverse MR analyses, SNPs with *P*<1×10^-5^ were considered significant. The following procedures was similar to the above one.

### 2.4 Statistical analyses

Exposure and outcome datasets were harmonized to ensure consistency between exposure and outcome alleles (37).To investigate the causal effect between TD and AA, the inverse variance weighting (IVW) method was used as the main method, and MR Egger, weighted median, simple mode and weighted mode were used as important supplements. Additionally, reverse-direction MR was conducted to evaluate the potential reverse causal association of TD on AA.

We used several sensitivity analyses to examine and correct causal estimates. Firstly, heterogeneity was assessed by Cohran Q test (38), When the *P*-value of the heterogeneity test was lower than 0.05, it indicated heterogeneity. Then, Mendelian Randomization Pleiotropy Residual Sum and Outlier (MR-PRESSO) technique was used to detect and remove heterogeneous SNPs (39). Besides, we also used MR-Egger intercept and funnel plots to assess horizontal pleiotropy. Sensitivity analysis was carried out based on the leave-one-out method.

All statistical analyses were conducted using the “Two-Sample MR” (version 0.5.9) packages in R version 4.0.3. Statistical significance was defined as a *P*-value less than 0.05.

## 3 Results

In summary, according to the specified selection criteria, the datasets included in this study were all published in the most recent time and in European populations, as detailed in **Supplementary Table S1**.

### 3.1 The causal effect of TD on AA via forward MR

**Figure 2:**
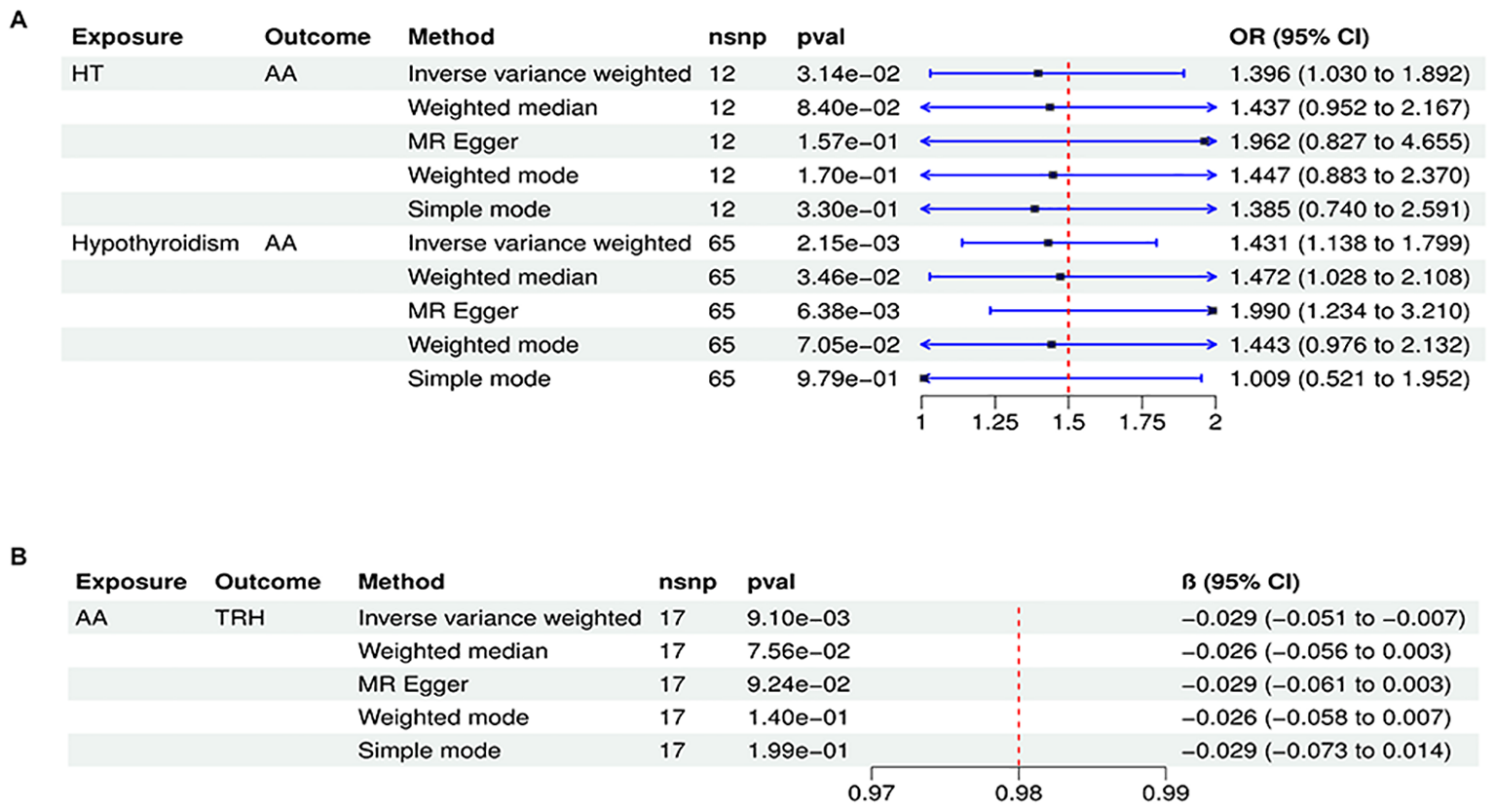
Significant causal plot between TD and AA in MR analysis. **(A)** Significant MR analysis of the causal effects of HT/Hypothyroidism on AA. **(B)** Significant MR analysis of the causal effects of AA on TRH.

**Figure 3:**
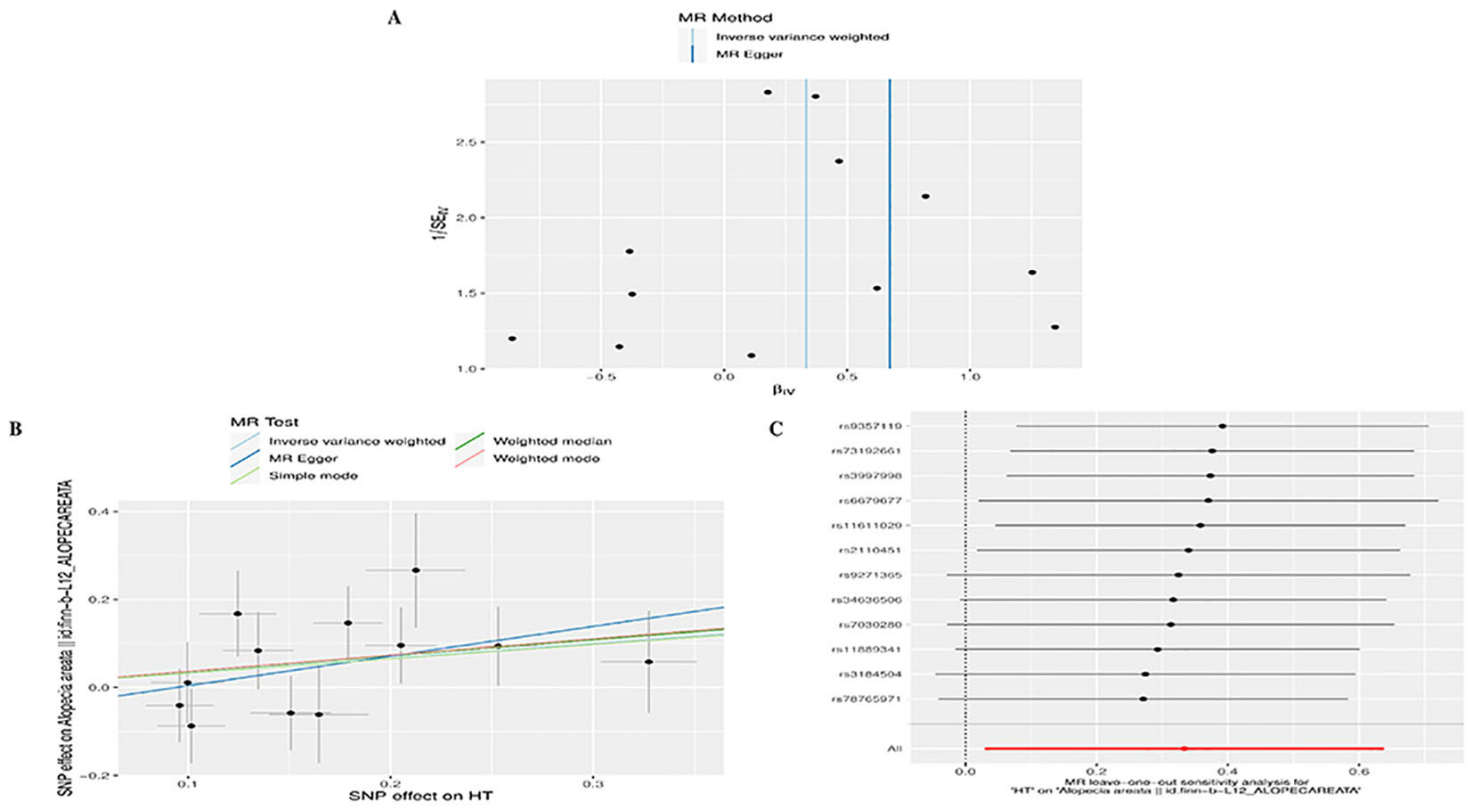
Plots of MR estimates of the causal relationship between HT and AA. **(A)** Leave-one-out sensitivity analysis of the association of HT on AA. **(B)** Scatter plot of the association of HT on AA. **(C)** Funnel plot of the association of HT on AA.

**Figure 4:**
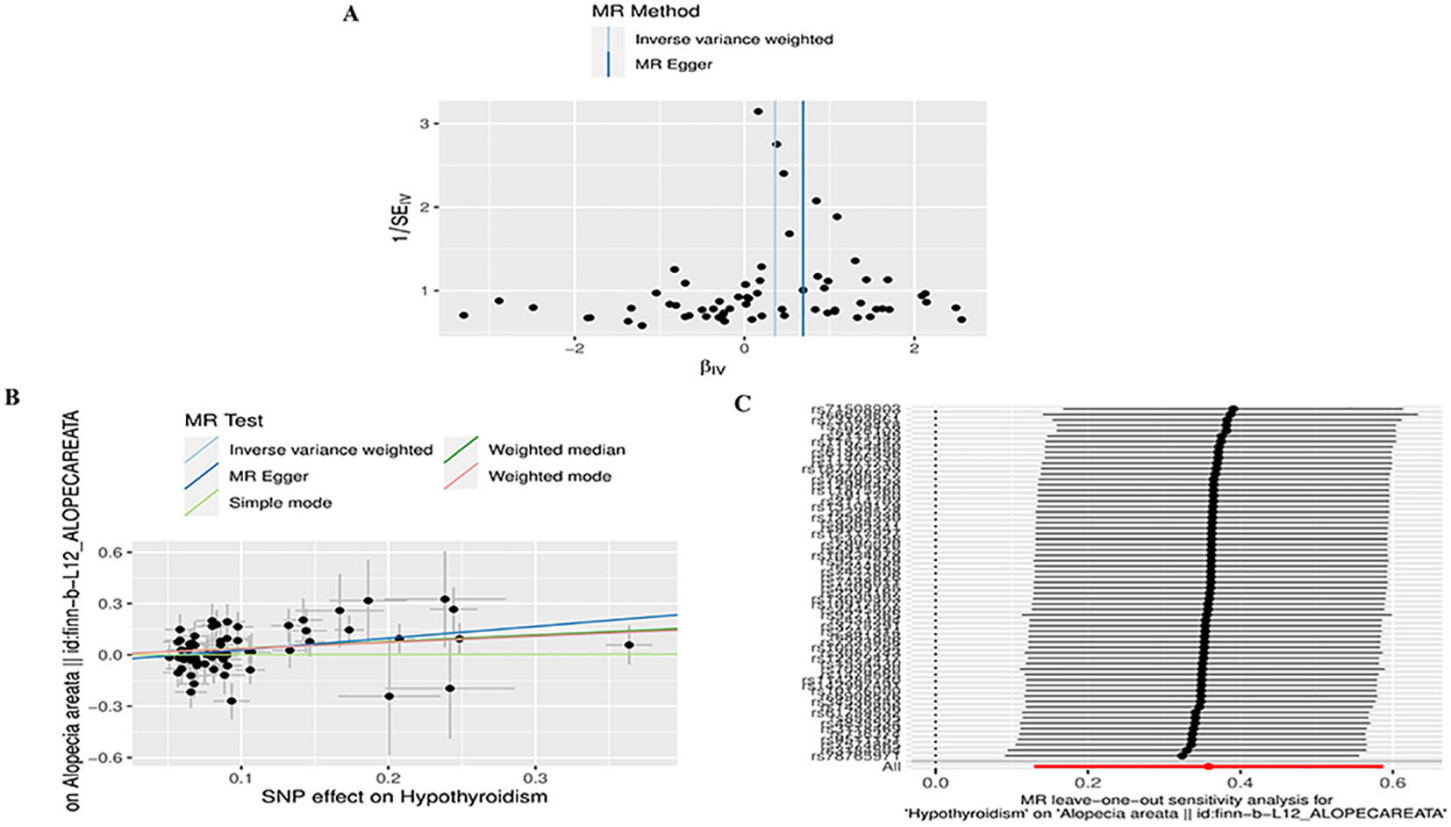
Plots of MR estimates of the causal relationship between Hypothyroidism and AA. **(A)** Funnel plot of the association of Hypothyroidism on AA. **(B)** Scatter plot of the association of Hypothyroidism on AA. **(C)** Leave-one-out sensitivity analysis of the association of Hypothyroidism on AA.

To conduct a comprehensive assessment of the association between TD and the likelihood of AA development, MR analyses were employed to validate associations with a statistically significant *P*-value of less than 0.05. After removing palindromic SNPs and outliers, we obtained 26, 12, 65,11,258,22,26,16,20, and 23 SNPs for GD, HT, hypothyroidism, hyperthyroidism, TC, TSH, TRH, THRα, TP and TG, respectively. **Supplementary Table S2** contains SNP information for all instrumental variables in forward and reverse MR analyses. We observed a significant causal relationship between TD and AA using the IVW method (HT and AA, IVW OR = 1.396, 95% CI 1.030-1.892, *P*=0.031; Hypothyroidism and AA, IVW OR = 1.431, 95% CI 1.138-1.799, *P*=0.002) (**Figure 2A**) (**Supplementary Table S3**). MR-Egger and Weight median were estimated in the same direction as the IVW method (HT and AA, *P*=0.157, *P*=0.084; hypothyroidism and AA, *P* = 0.006, *P*=0.03), despite not significant (**Figure 2A**) (**Supplementary Table S3**). The reliability of the MR analysis results can be further substantiated by employing the Leave-one-out method of sensitivity analyses to ascertain the impact of individual genetic variants on the overall outcomes (**Figures 3C**, **4C**). The absence of significant horizontal pleiotropy was indicated by the MR-pleiotropy test and MR-Egger regression (HT for AA, *P*=0.428; Hypothyroidism for AA, *P*=0.129) (**Figures 3B**, **4B**) (**Supplementary Table S4**). The Cochran’s Q test and MR-PRESSO global test suggested no evidence of heterogeneity (HT on AA, Cochran’s Q=11.13, *P*=0.432, *P* =0.49; Hypothyroidism on AA, Cochran’s Q=64, *P*=0.339, *P*=0.354) (**Figures 3A**, **4A**) (**Supplementary Table S4**). In addition, the summary data from IVW analysis showed no evidence of a causal relationship between GD (*P*=0.398), Hyperthyroidism (*P*=0.974), TC (*P*=0.669), TSH (*P*=0.847), TRH (*P*=0.386), THRα (*P*=0.942), TP (*P*=0.828), TG (*P*=0.460), and AA (**Supplementary Table S3**). In addition, the results of funnel plot, leave-one-out analysis and forest plot of forward MR analysis in this study were presented in **Supplementary Figures S1**, **S2**. It is worth mentioning that according to our screening criteria, SNPs strongly associated with TBG were not screened out, so we did not establish a causal relationship between TBG and AA.

### 3.2 The causal effect of AA on TD via reverse MR

**Figure 5:**
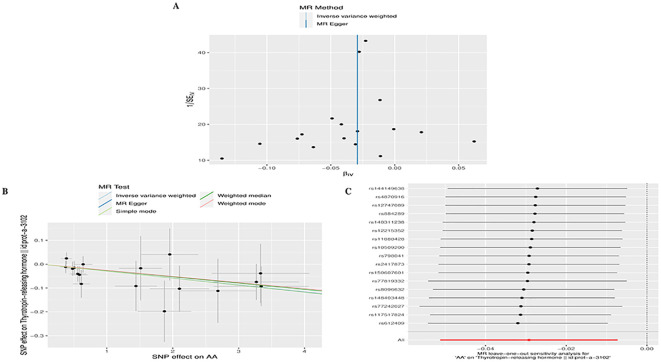
Plots of MR estimates of the causal relationship between AA and TRH. **(A)** Leave-one-out sensitivity analysis of the association of AA on TRH. **(B)** Scatter plot of the association of AA on TRH. **(C)** Funnel plot of the association of AA on TRH.

In reverse MR analysis, we considered AA as an exposure and TD as outcome. As a result of SNP filtering, 18, 18, 18,18,5,17,17,17,17,17 and 4 SNPs were included for GD, HT, hypothyroidism, hyperthyroidism, TC, TSH, TRH, THRα, TP, TG and TBG, respectively, in final analysis (**Supplementary Table S2**). According to **Figure 2B**, IVW findings suggested that the existence of AA was linked to a reduction in TRH levels (AA on TRH, β=-0.029, 95%CI=-0.051 to -0.007, *P*=0.009). The Leave-one-out method was used to assess robustness of these results (**Figure 5C**). MR pleiotropy trials and MR Egger regression were performed to assess horizontal pleiotropy, and there was no evidence of horizontal pleiotropy (AA for TRH, *P*=0.998) (**Figure 5B**) (**Supplementary Table S4**). The Cochran’s Q test and MR-PRESSO global test suggested no evidence of heterogeneity (AA on TRH, Cochran’s Q=7.47, *P*=0.963, Global Test’*P* =0.969) (**Figure 5A**) (**Supplementary Table S4**). MR analysis showed that there had no causal association between AA and GD, HT, Hypothyroidism, Hyperthyroidism, TC, TSH, TBG, THRα, TP, TG and TBG (**Supplementary Table S3**). Leave-one-out, scatter plot, and funnel plots of the results of the reverse MR analysis are shown in **Supplementary Figures S3**-**S5**, respectively.

### 3.3 Confounding analysis

Through forward and reverse MR analysis, our study found that HT and hypothyroidism had causal effects on AA. To ensure that the instrumental variables (IVs) associated with hypothyroidism and HT were independent of confounding factors, we examined the autonomy of our selected SNPs from common risk factors for AA. Specifically, we assessed their associations with recognized risk factors such as genetic factors, mental stress and intestinal dysbiosis. In hypothyroidism, we found that rs12593201, rs434294 and rs9271365 were associated with AA risk factors such as depression, insomnia and sleep disorders. No suspicious SNP was identified in HT. Re-analysis with exclusion of three SNPs demonstrated that our estimates remain significant: hypoglycemia: (IVW OR = 1.431, 95% CI 1.138-1.799, *P*=0.002).

## 4 Discussion

Our two-sample MR study provides novel evidence for a causal relationship between thyroid dysfunction and AA. Our study found that HT and hypothyroidism have a causal effect on AA, suggesting that HT and hypothyroidism themselves may play a causal role in the pathogenesis of AA. The results of two recently published MR studies on AA and hypothyroidism are consistent with our findings (18, 40), but they only explored the causal relationship between hypothyroidism and AA, the sample size of the hypothyroidism GWAS data was not the most up-to-date and comprehensive, and it did not cover other thyroid disease and relevant measures of thyroid function. However, there was no causal relationship between GD, hyperthyroidism, TC, TSH, TRH, TBG, THRα, TP, TG, and AA. In addition, reverse MR analysis showed that genetic susceptibility to AA may affect the risk of TRH. A series of sensitive analyses support these findings.

Autoimmune thyroid disease (AITD) is the most common organ-specific autoimmune disease (41). The AITD spectrum includes Graves-Basedow disease (GBD) and Hashimoto’s thyroiditis (HT) as well as a wide range of clinical manifestations, ranging from Hashimoto’s overt or subclinical hypothyroidism, euthyroidism, to Graves’ subclinical or overt hyperthyroidism (42). According to our search, the first case of hypothyroidism with AA was reported in 1960. Alopecia areata (AA) is often associated with other autoimmune diseases, such as thyroid disease (43), asthma, allergic rhinitis (44), and other skin diseases (45) with autoimmune causes. Thyroid dysfunctions resulting from the autoimmune mechanism are the leading disorders reported in the literature related to AA patients (15, 46). Several descriptive studies have investigated the association between AA and thyroid dysfunction in the form of prevalence, comorbidities, and clinical significance (13, 16, 43, 47–49). Recently, a new systematic review and meta-analysis of AA-related medical comorbidities showed that Hashimoto ‘s thyroiditis (OR 4.31,95% CI 2.51-7.40) was one of the comorbidities with a higher odds ratio in AA patients compared with healthy controls (50).

Previous studies have shown that hypothyroidism is associated with an increased risk of AA (51, 52). A retrospective study of 78 newly diagnosed alopecia areata who presented to community dermatology clinics between 2007 and 2011 found that 13 cases of AA (16.6%) had hypothyroidism (53). A prospective study of women in the United States from 2002 to 2014 found that a history of hypothyroidism was associated with an increased risk of AA (54). A study comparing the clinical patterns of 89 patients with alopecia areata found that approximately 25% of patients with AA had comorbid thyroid lesions, the most common of which was hypothyroidism (n=20) (55).

A meta-analysis by Kinoshita-Ise et al. (56) found that compared with healthy controls, positive anti-thyroid peroxidase antibody (TPO-Ab) (OR = 3.58; 95% CI 1.96–6.53) and anti-thyroglobulin antibody (TG-Ab) (OR = 4.44; 95% CI 1.54–12.75) were more common in AA patients. Moreover, the risk of both or either of TPO-Ab and TG-Ab being positive in the same AA patient was OR = 2.32 (95% CI 1.08–4.98) and OR = 6.34 (95% CI 2.24–17.93), respectively. In addition, the study also found that the proportion of TSH (thyroid stimulating hormone) receptor antibody (TR-Ab) positive patients in AA patients was higher (OR = 60.90; 95% CI 34.61-107.18).

Previous studies have observed a correlation between thyroid disease and AA, but the causal and biological links between the two are unclear. AA shares an autoimmune background with autoimmune thyroid disease, either sporadic or autoimmune polyglandular syndrome (52). The cause of AA is unknown; however, genetic susceptibility, different types of autoimmunity, and, probably, stress are regarded as contributors (43, 57). In HT, autoimmune processes lead to apoptosis and destruction of thyroid follicles, and subsequent hypothyroidism. Thyroid hormones are essential for the growth and maintenance of hair follicles (58, 59). In hypothyroid patients, the epidermis is thin, and they frequently develop alopecia (60), showing that thyroid hormone signaling can regulate both skin proliferation and hair growth (61, 62). HT is currently the leading cause of primary hypothyroidism, both in adolescents and adults. It is a T-cell-mediated autoimmune disorder characterized by thyroid lymphocytic infiltration (63). T lymphocytes cause hair destruction in AA, hence T lymphocytes and associated cytokines that infiltrate the areas around the hair follicles play a major part in the disease’s etiology. Cytotoxic CD8+NKG2D+ T lymphocytes are the primary immunocytes that infiltrate the surroundings of HFs and are held to be the key cells that drive the disease pathogenesis NKG2D is an activating receptor expressed on CD8+ T cells and NK cells which recognize NKG2D ligands, like ULBP3/6 and MICA, and then upregulate MHC expression, which is crucial in mediating HP-IP collapse (64). HLA (Human Leucocytes Antigens) represents another link between AA and autoimmune thyroid disease. Some data showed that HLA-DQB1*03 is connected to both AA and antibodies-induced hypothyroidism. A genetic association study of HLA genes also found that the DRB1*15: 01 DQB1*06: 02 haplotype frequency was considerably greater in TR-Ab-positive individuals diagnosed with AA versus controls (65). Although the etiopathogenesis of AA has yet to be fully elucidated, its current understanding includes the genetic factors and various environmental triggers, whose interaction influences the autoreactive cytotoxic T-lymphocyte activation and increased secretion of interferon (IFN)-γ in a predisposed individual. Type 1 inflammatory response leads to the loss of the hair follicle immune privilege, overexpression of MHC class I, and consequent autoimmune assault on hair follicles (66). In summary, previous studies support the causal relationship between HT and hypothyroidism and alopecia areata observed in our current study. Of course, not all patients with HT and hypothyroidism will have alopecia areata. This may be due to individual differences, disease severity, treatment measures, and other factors. In clinical practice, we recommend routine screening of thyroid function in patients with alopecia areata, and further identify prevention and treatment methods.

Our results have certain significance for the diagnosis and management of TD and AA patients. First, our study is the largest and most comprehensive MR study of TD and AA to date, assessing the causal relationship between TD and AA and minimizing potential confounders. Secondly, our findings provide new insights into the occurrence of AA in TD patients, provide guidance for the treatment of AA patients, and help improve the quality of life of AA patients. Consistent monitoring of thyroid-related hormones and prompt diagnosis and treatment of TD can provide valuable insights for the management of AA and lifestyle interventions. In addition, when treating patients with AA, it is important to pay attention to the effect that the drug may have on thyroid function. Furthermore, future research should focus on establishing the pathogenesis between TD and AA, and explore novel effective biomarkers that affect the pathogenesis of AA.

Our studies also have some limitations. Firstly, due to the limited data available for GWAS in alopecia areata, this study failed to perform a confirmatory analysis. Secondly, our research population was all European, and the conclusions of the study may not be applicable to other ethnic groups. Finally, although our study provides genetic evidence for causality, additional research is needed to further elucidate the underlying mechanisms.

## 5 Conclusion

In conclusion, our study suggests that HT and hypothyroidism can lead to AA. Based on the results of our study, we recommend thyroid function and related antibody tests for the clinical treatment of patients with AA. Prompt treatment of HT and hypothyroidism may reduce the incidence of AA. In addition, the identification of a potential causal relationship between HT and hypothyroidism and AA provides a new avenue for studying the origin and progression of AA.

## Data Availability

The original contributions presented in the study are included in the article/**Supplementary Material**. Further inquiries can be directed to the corresponding author.

## Supplementary material

Supplementary Figure 1 Funnel plot, Scatter plot, Leave-one-out sensitivity analysis of the association of GD **(A–C)**, hyperthyroidism **(D–F)**, TC **(G–I)**, TSH **(J–L)** on AA. GD, Graves’ disease; TC, Thyroid cancer; TSH, Thyroid Stimulating Hormone; AA, alopecia areata.

Supplementary Figure 2 Funnel plot, Scatter plot, Leave-one-out sensitivity analysis of the association of TRH **(A–C)**, THRα **(D–F)**, TP **(G–I)**, TG **(J–L)** on AA. TRH, Thyrotropin-releasing hormone; THRα, Thyroid hormone receptor alpha; TP, Thyroid peroxidase; TG, Thyroglobulin; AA, alopecia areata.

Supplementary Figure 3 Funnel plot of the association of AA on GD **(A)**, HT **(B)**, hypothyroidism**(C)**, hyperthyroidism **(D)**, TC **(E)**, TSH **(F)**, THRα**(G)**, TP **(H)**, TG**(I)**, TBG**(J)**. AA, alopecia areata; GD, Graves’ disease; HT, Hashimoto’s thyroiditis; TC, Thyroid cancer; TSH, Thyroid Stimulating Hormone; THRα, Thyroid hormone receptor alpha; TP, Thyroid peroxidase; TG, Thyroglobulin; TBG, Thyroxine-Binding Globulin.

Supplementary Figure 4 Scatter plot of the association of AA on GD **(A)**, HT **(B)**, hypothyroidism **(C)**, hyperthyroidism **(D)**, TC **(E)**, TSH **(F)**, THRα **(G)**, TP **(H)**, TG **(I)**, TBG **(J)**. AA, alopecia areata; GD, Graves’ disease; HT, Hashimoto’s thyroiditis; TC, Thyroid cancer; TSH, Thyroid Stimulating Hormone; THRα, Thyroid hormone receptor alpha; TP, Thyroid peroxidase; TG, Thyroglobulin; TBG, Thyroxine-Binding Globulin.

Supplementary Figure 5 Leave-one-out sensitivity analysis of the association of AA on GD **(A)**, HT **(B)**, hypothyroidism **(C)**, hyperthyroidism **(D)**, TC **(E)**, TSH **(F)**, THRα **(G)**, TP **(H)**, TG**(I)**, TBG**(J)**. AA, alopecia areata; GD, Graves’ disease; HT, Hashimoto’s thyroiditis; TC, Thyroid cancer; TSH, Thyroid Stimulating Hormone; THRα, Thyroid hormone receptor alpha; TP, Thyroid peroxidase; TG, Thyroglobulin; TBG, Thyroxine-Binding Globulin.
