# Supplementary material for: The genetic link between thyroid dysfunction and alopecia areata: a bidirectional two-sample Mendelian randomization study
Source: Front Endocrinol (Lausanne). 2024 Aug 14;15:1440941. doi: 10.3389/fendo.2024.1440941 (PMC11349512; doi:10.3389/fendo.2024.1440941)
Supplement: Supplementary file 1 [file Datasheet1.docx]

***An overview of supplementary figures and supplementary tables***

**Supplementary Table S1**: Baseline characteristics of TD and AA dataset in the present study.

**Supplementary Table S2**: SNP information for all tool variables in forward and reverse MR analysis.

**Supplementary Table S3**: MR Results between TD and AA.

**Supplementary Table S4**: Pleiotropy and heterogeneity test between TD and AA.

**Supplementary Table S5**: STROBE-MR checklist of recommended items to address in reports of Mendelian randomization studies.

**Supplementary Figure S1**: Funnel plot, Scatter plot, Leave-one-out sensitivity analysis of the association of GD(A)(B)(C), hyperthyroidism(D)(E)(F), TC(G)(H)(I), TSH(J)(K)(L) on AA. GD, Graves’ disease; TC, Thyroid cancer; TSH, Thyroid Stimulating Hormone; AA, alopecia areata.

**Supplementary Figure S2**: Funnel plot, Scatter plot, Leave-one-out sensitivity analysis of the association of TRH(A)(B)(C), THRɑ(D)(E)(F), TP(G)(H)(I), TG(J)(K)(L) on AA. TRH, Thyrotropin-releasing hormone; THRɑ, Thyroid hormone receptor alpha; TP, Thyroid peroxidase; TG, Thyroglobulin; AA, alopecia areata.

**Supplementary Figure S3**: Funnel plot of the association of AA on GD (A), HT (B), hypothyroidism(C), hyperthyroidism (D), TC (E), TSH (F), THRɑ(G), TP (H), TG(I), TBG(J). AA, alopecia areata; GD, Graves’ disease; HT, Hashimoto's thyroiditis; TC, Thyroid cancer; TSH, Thyroid Stimulating Hormone; THRɑ, Thyroid hormone receptor alpha; TP, Thyroid peroxidase; TG, Thyroglobulin; TBG, Thyroxine-Binding Globulin.

**Supplementary Figure S4**: Scatter plot of the association of AA on GD (A), HT (B), hypothyroidism(C), hyperthyroidism (D), TC (E), TSH (F), THRɑ(G), TP (H), TG(I), TBG(J). AA, alopecia areata; GD, Graves’ disease; HT, Hashimoto's thyroiditis; TC, Thyroid cancer; TSH, Thyroid Stimulating Hormone; THRɑ, Thyroid hormone receptor alpha; TP, Thyroid peroxidase; TG, Thyroglobulin; TBG, Thyroxine-Binding Globulin.

**Supplementary Figure S5**: Leave-one-out sensitivity analysis of the association of AA on GD (A), HT (B), hypothyroidism(C), hyperthyroidism (D), TC (E), TSH (F), THRɑ(G), TP (H), TG(I), TBG(J). AA, alopecia areata; GD, Graves’ disease; HT, Hashimoto's thyroiditis; TC, Thyroid cancer; TSH, Thyroid Stimulating Hormone; THRɑ, Thyroid hormone receptor alpha; TP, Thyroid peroxidase; TG, Thyroglobulin; TBG, Thyroxine-Binding Globulin.
