# Supplementary figures and images for: The genetic link between thyroid dysfunction and alopecia areata: a bidirectional two-sample Mendelian randomization study
Source: Front Endocrinol (Lausanne). 2024 Aug 14;15:1440941. doi: 10.3389/fendo.2024.1440941 (PMC11349512; doi:10.3389/fendo.2024.1440941)

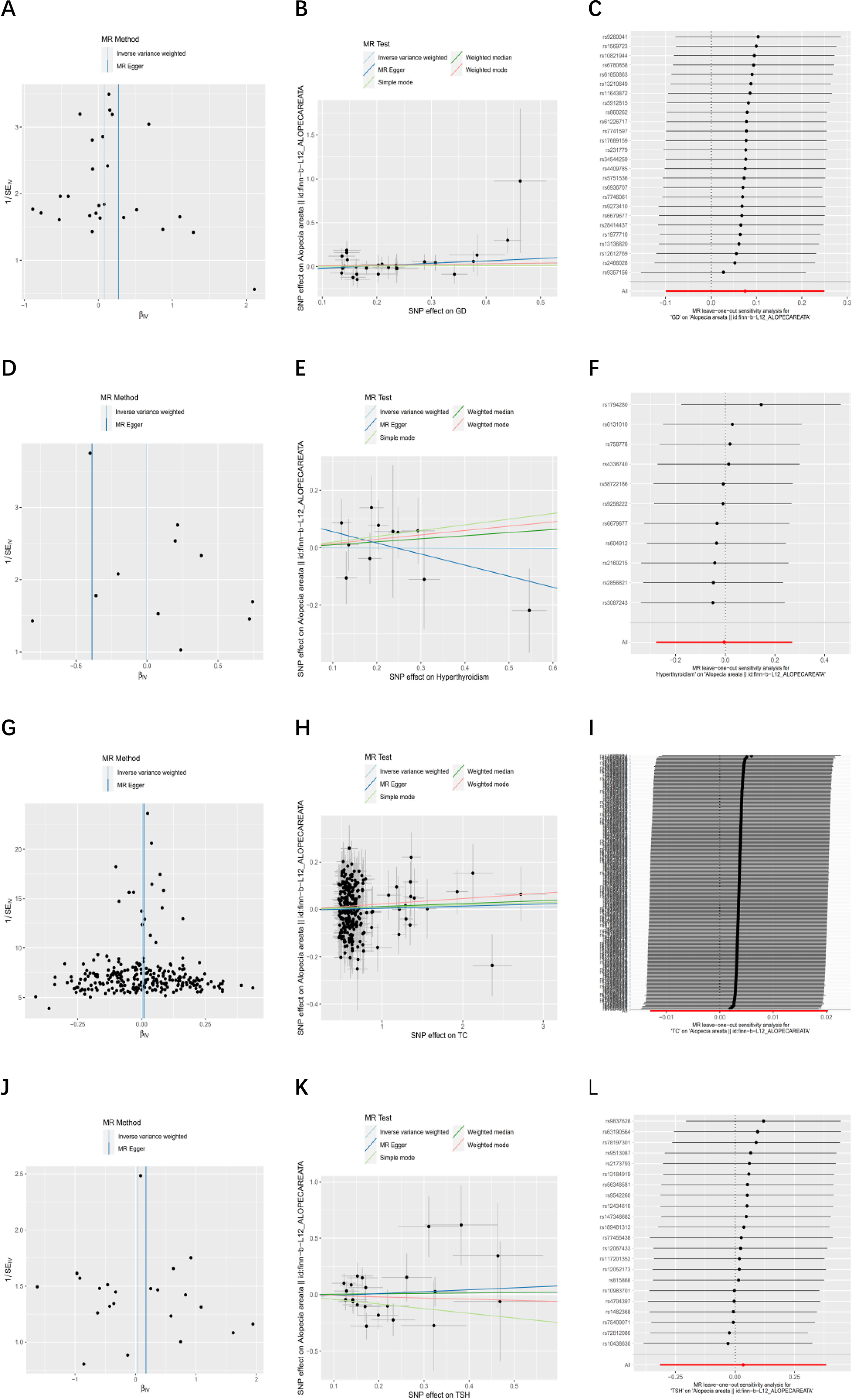

Supplement: Supplementary Figure 1 — Funnel plot, Scatter plot, Leave-one-out sensitivity analysis of the association of GD (A–C), hyperthyroidism (D–F), TC (G–I), TSH (J–L) on AA. GD, Graves’ disease; TC, Thyroid cancer; TSH, Thyroid Stimulating Hormone; AA, alopecia areata. [file Image1.tif]

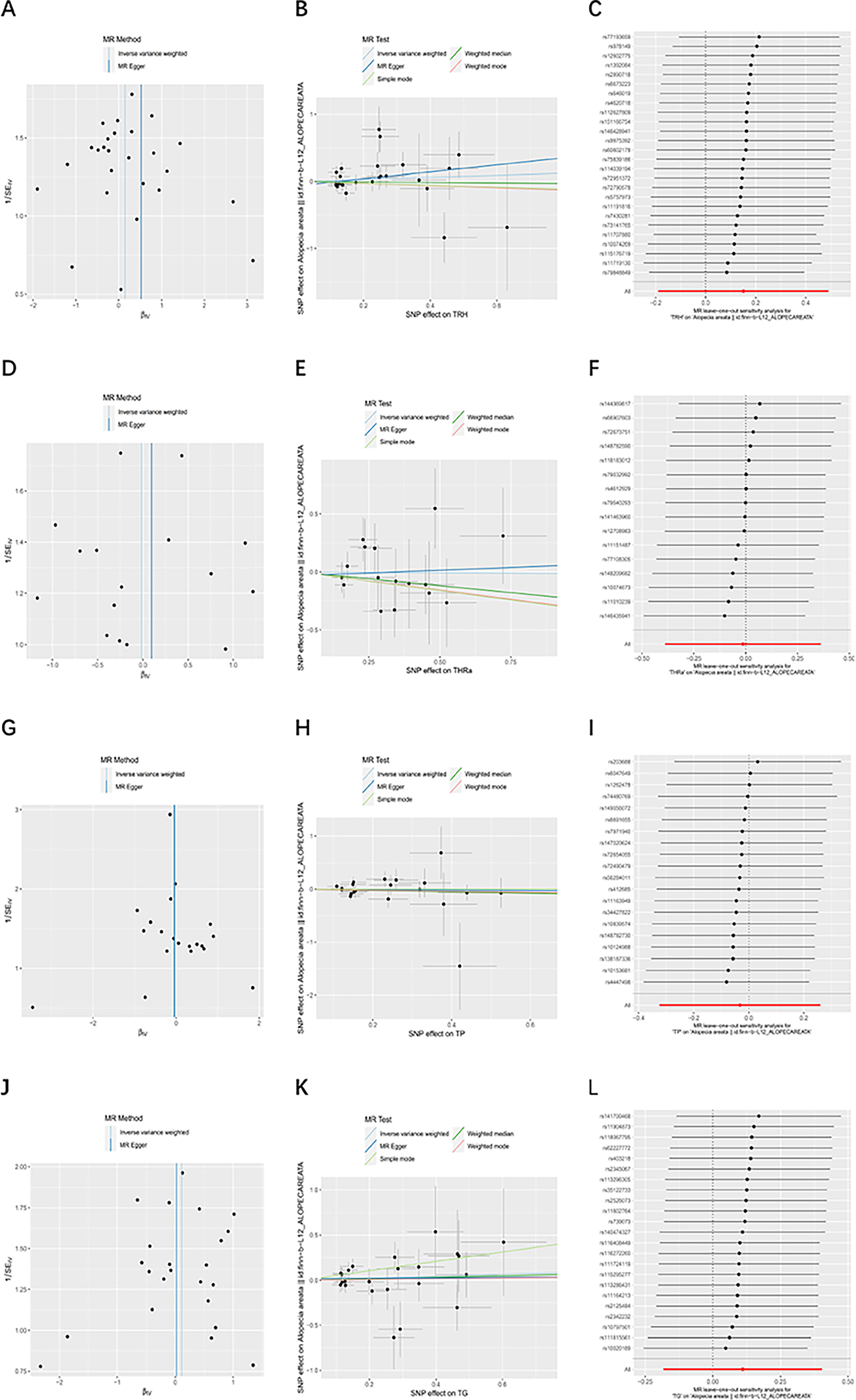

Supplement: Supplementary Figure 2 — Funnel plot, Scatter plot, Leave-one-out sensitivity analysis of the association of TRH (A–C), THRα (D–F), TP (G–I), TG (J–L) on AA. TRH, Thyrotropin-releasing hormone; THRα, Thyroid hormone receptor alpha; TP, Thyroid peroxidase; TG, Thyroglobulin; AA, alopecia areata. [file Image2.tif]

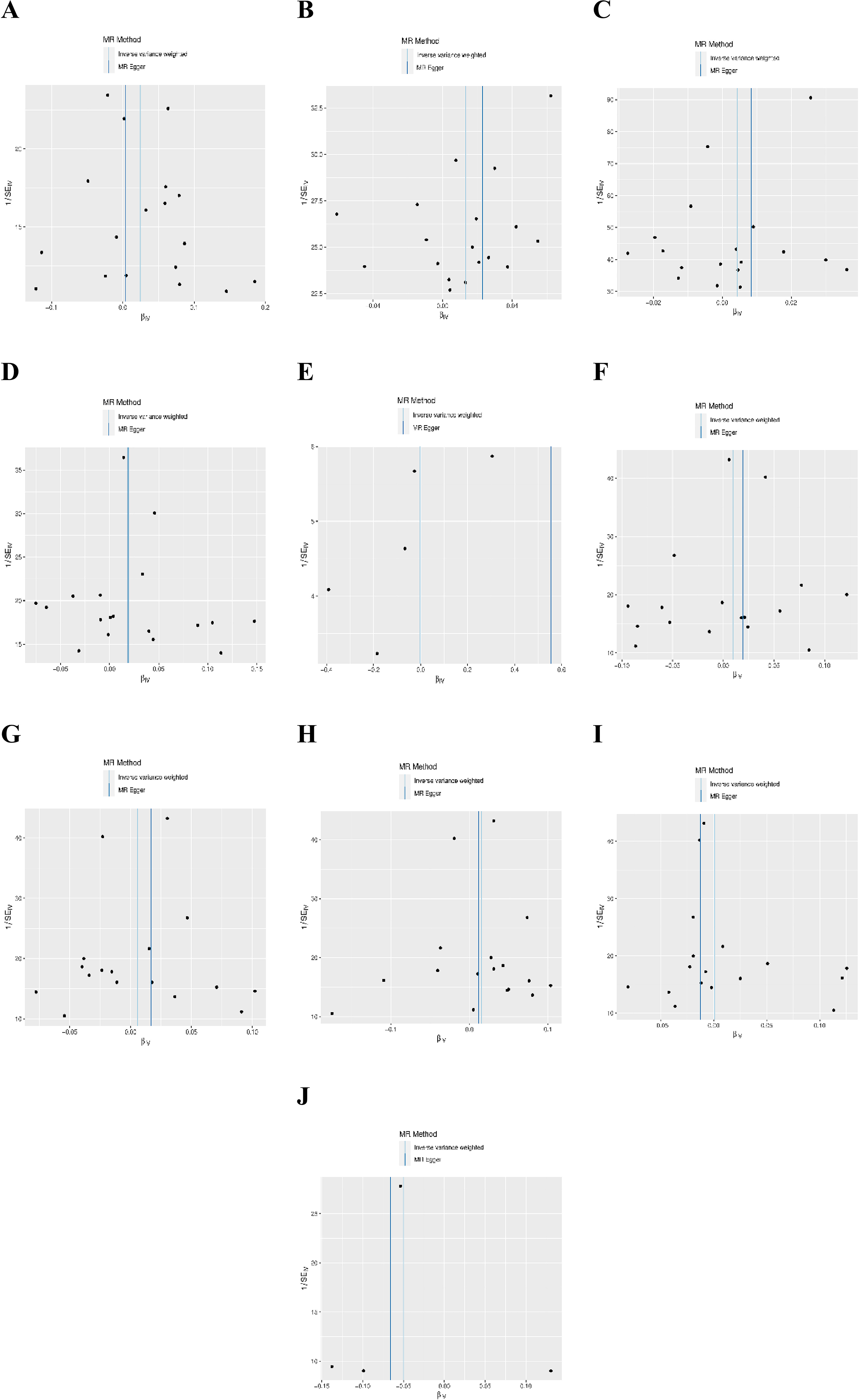

Supplement: Supplementary Figure 3 — Funnel plot of the association of AA on GD (A), HT (B), hypothyroidism(C), hyperthyroidism (D), TC (E), TSH (F), THRα(G), TP (H), TG(I), TBG(J). AA, alopecia areata; GD, Graves’ disease; HT, Hashimoto’s thyroiditis; TC, Thyroid cancer; TSH, Thyroid Stimulating Hormone; THRα, Thyroid hormone receptor alpha; TP, Thyroid peroxidase; TG, Thyroglobulin; TBG, Thyroxine-Binding Globulin. [file Image3.tif]

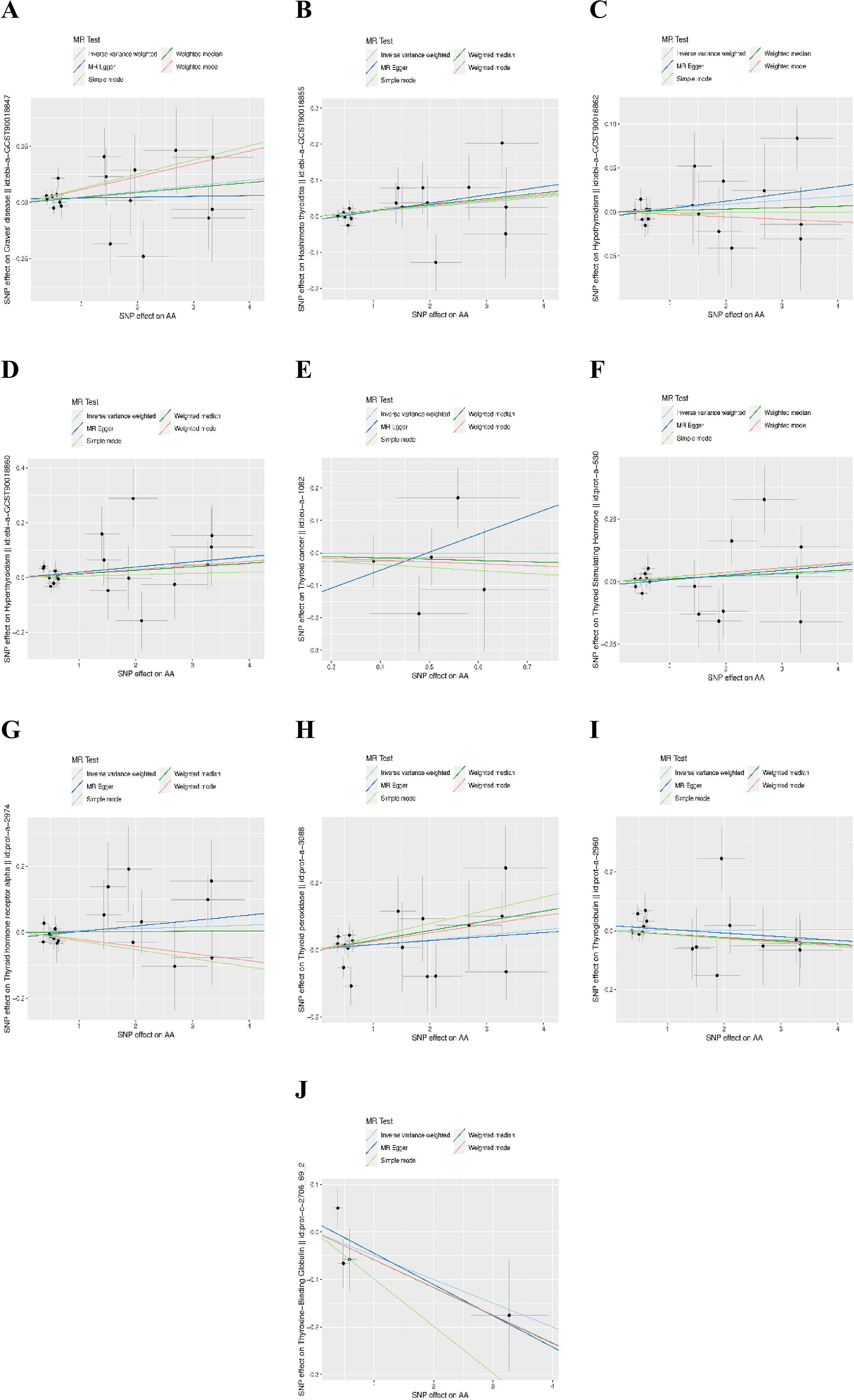

Supplement: Supplementary Figure 4 — Scatter plot of the association of AA on GD (A), HT (B), hypothyroidism (C), hyperthyroidism (D), TC (E), TSH (F), THRα (G), TP (H), TG (I), TBG (J). AA, alopecia areata; GD, Graves’ disease; HT, Hashimoto’s thyroiditis; TC, Thyroid cancer; TSH, Thyroid Stimulating Hormone; THRα, Thyroid hormone receptor alpha; TP, Thyroid peroxidase; TG, Thyroglobulin; TBG, Thyroxine-Binding Globulin. [file Image4.tif]

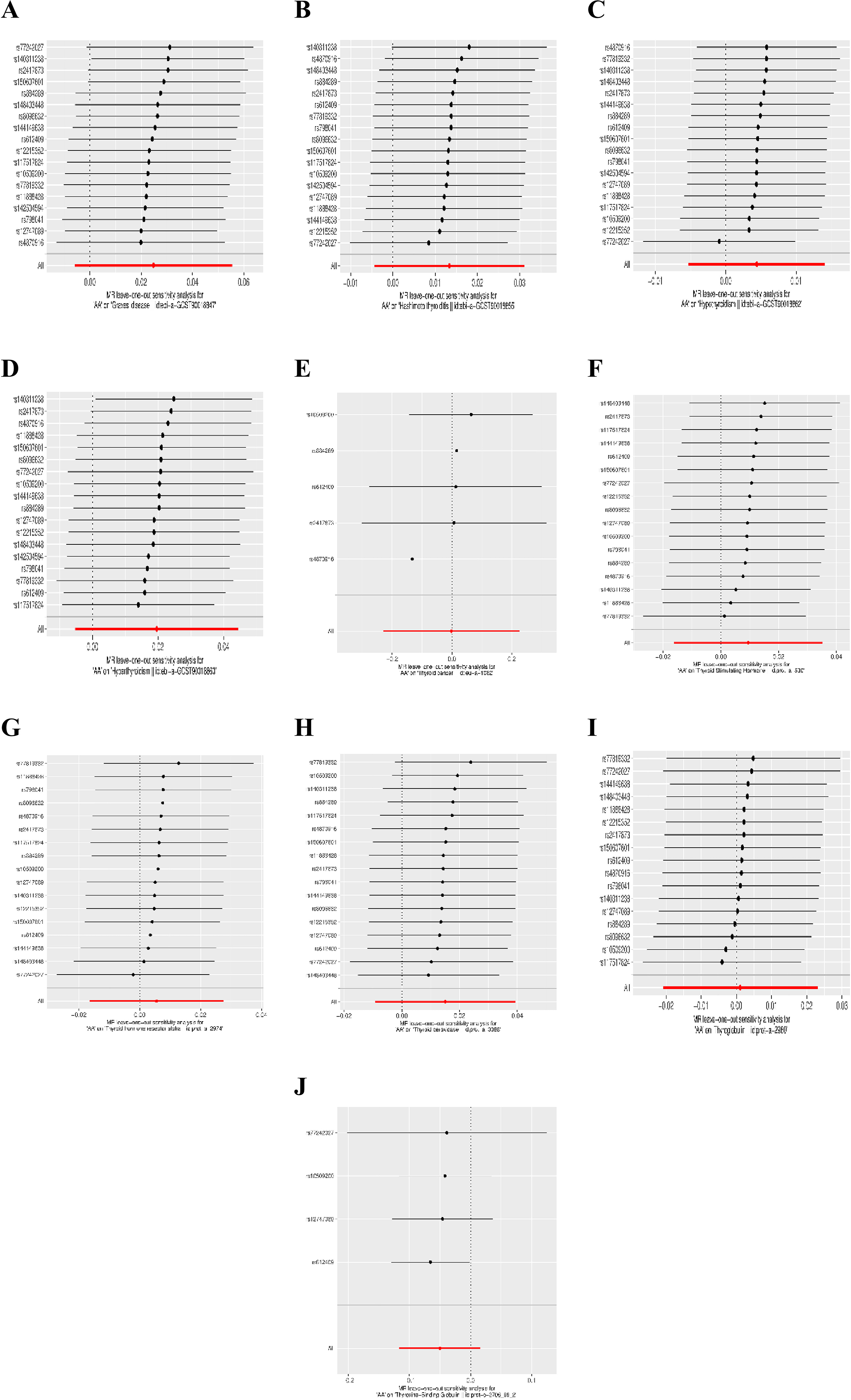

Supplement: Supplementary Figure 5 — Leave-one-out sensitivity analysis of the association of AA on GD (A), HT (B), hypothyroidism (C), hyperthyroidism (D), TC (E), TSH (F), THRα (G), TP (H), TG(I), TBG(J). AA, alopecia areata; GD, Graves’ disease; HT, Hashimoto’s thyroiditis; TC, Thyroid cancer; TSH, Thyroid Stimulating Hormone; THRα, Thyroid hormone receptor alpha; TP, Thyroid peroxidase; TG, Thyroglobulin; TBG, Thyroxine-Binding Globulin. [file Image5.tif]
