# Supplementary material for: The genetic link between thyroid dysfunction and alopecia areata: a bidirectional two-sample Mendelian randomization study
Source: Front Endocrinol (Lausanne). 2024 Aug 14;15:1440941. doi: 10.3389/fendo.2024.1440941 (PMC11349512; doi:10.3389/fendo.2024.1440941)
Supplement: Supplementary file 7 [file Table1.docx]

***An overview of supplementary figures and supplementary tables***

**Supplementary Table S1**: Baseline characteristics of TD and AA dataset in the present study.

**Supplementary Table S2**: SNP information for all tool variables in forward and reverse MR analysis.

**Supplementary Table S3**: MR Results between TD and AA.

**Supplementary Table S4**: Pleiotropy and heterogeneity test between TD and AA.

**Supplementary Table S5**: STROBE-MR checklist of recommended items to address in reports of Mendelian randomization studies.

**Supplementary Figure S1**: Funnel plot, Scatter plot, Leave-one-out sensitivity analysis of the association of GD(A)(B)(C), hyperthyroidism(D)(E)(F), TC(G)(H)(I), TSH(J)(K)(L) on AA. GD, Graves’ disease; TC, Thyroid cancer; TSH, Thyroid Stimulating Hormone; AA, alopecia areata.

**Supplementary Figure S2**: Funnel plot, Scatter plot, Leave-one-out sensitivity analysis of the association of TRH(A)(B)(C), THRɑ(D)(E)(F), TP(G)(H)(I), TG(J)(K)(L) on AA. TRH, Thyrotropin-releasing hormone; THRɑ, Thyroid hormone receptor alpha; TP, Thyroid peroxidase; TG, Thyroglobulin; AA, alopecia areata.

**Supplementary Figure S3**: Funnel plot of the association of AA on GD (A), HT (B), hypothyroidism(C), hyperthyroidism (D), TC (E), TSH (F), THRɑ(G), TP (H), TG(I), TBG(J). AA, alopecia areata; GD, Graves’ disease; HT, Hashimoto's thyroiditis; TC, Thyroid cancer; TSH, Thyroid Stimulating Hormone; THRɑ, Thyroid hormone receptor alpha; TP, Thyroid peroxidase; TG, Thyroglobulin; TBG, Thyroxine-Binding Globulin.

**Supplementary Figure S4**: Scatter plot of the association of AA on GD (A), HT (B), hypothyroidism(C), hyperthyroidism (D), TC (E), TSH (F), THRɑ(G), TP (H), TG(I), TBG(J). AA, alopecia areata; GD, Graves’ disease; HT, Hashimoto's thyroiditis; TC, Thyroid cancer; TSH, Thyroid Stimulating Hormone; THRɑ, Thyroid hormone receptor alpha; TP, Thyroid peroxidase; TG, Thyroglobulin; TBG, Thyroxine-Binding Globulin.

**Supplementary Figure S5**: Leave-one-out sensitivity analysis of the association of AA on GD (A), HT (B), hypothyroidism(C), hyperthyroidism (D), TC (E), TSH (F), THRɑ(G), TP (H), TG(I), TBG(J). AA, alopecia areata; GD, Graves’ disease; HT, Hashimoto's thyroiditis; TC, Thyroid cancer; TSH, Thyroid Stimulating Hormone; THRɑ, Thyroid hormone receptor alpha; TP, Thyroid peroxidase; TG, Thyroglobulin; TBG, Thyroxine-Binding Globulin.

***Supplementary Table S1*:** Baseline characteristics of TD and AA dataset in the present study.

| **Traits** | **Dataset** | **Author** | **Population** | **Sample size** | **n case** | **n control** | **Sex** | **n SNPs** | **Year** | **PMID** |
| --- | --- | --- | --- | --- | --- | --- | --- | --- | --- | --- |
| AITD |  |  |  |  |  |  |  |  |  |  |
| GD | ebi-a-GCST90018847 | Sakaue S | European | 458620 | 1678 | 456942 | NA | 24189816 | 2021 | 34594039 |
| HT | ebi-a-GCST90018855 | Sakaue S | European | 395640 | 15654 | 379986 | NA | 24146037 | 2021 | 34594039 |
| Hypothyroidism | ebi-a-GCST90018862 | Sakaue S | European | 410141 | 30155 | 379986 | NA | 24138872 | 2021 | 34594039 |
| Hyperthyroidism | ebi-a-GCST90018860 | Sakaue S | European | 460499 | 3557 | 456942 | NA | 24189279 | 2021 | 34594039 |
| TC | ieu-a-1082 | Kohler A | European | 1080 | 649 | 431 | Males and Females | 572028 | 2013 | 23894154 |
| TSH | prot-a-530 | Sun BB | European | 3301 | NA | NA | Males and Females | 10534735 | 2018 | 29875488 |
| TRH | prot-a-3102 | Sun BB | European | 3301 | NA | NA | Males and Females | 10534735 | 2018 | 29875488 |
| THRα | prot-a-2974 | Sun BB | European | 3301 | NA | NA | Males and Females | 10534735 | 2018 | 29875488 |
| TP | prot-a-3088 | Sun BB | European | 3301 | NA | NA | Males and Females | 10534735 | 2018 | 29875488 |
| TG | prot-a-2960 | Sun BB | European | 3301 | NA | NA | Males and Females | 10534735 | 2018 | 29875488 |
| TBG | prot-c-2706_69_2 | Suhre K | European | NA | NA | NA | Males and Females | 501428 | 2019 | 28240269 |
| AA | finn-b-L12_ALOPECAREATA | NA | European | 211428 | 289 | 211139 | Males and Females | 16380450 | 2021 | NA |

AA, Alopecia areata; AITD, Autoimmune thyroid disease; SNP, single-nucleotide polymorphism; GD, Graves' disease; HT, Hashimoto's thyroiditis; TC, thyroid cancer; TSH, thyroid stimulating hormone; TRH, thyrotropin-releasing hormone, TBG, thyroxine-binding globulin; THRα, thyroid hormone receptor alpha; TP, thyroid peroxidase; TG, thyroglobulin.
