# Supplementary material for: The genetic link between thyroid dysfunction and alopecia areata: a bidirectional two-sample Mendelian randomization study
Source: Front Endocrinol (Lausanne). 2024 Aug 14;15:1440941. doi: 10.3389/fendo.2024.1440941 (PMC11349512; doi:10.3389/fendo.2024.1440941)
Supplement: Supplementary file 9 [file Table3.docx]

***Supplementary Table S3*:** MR Results between TD and AA

| **Exposure** | **Outcome** | **Method** | **SNP** | ***P* val** | **or/β** | **or_lci95/** **lo_ci** | | **or_uci95/** **up_ci** |
| --- | --- | --- | --- | --- | --- | --- | --- | --- |
| GD | AA | IVW | 26 | 0.398 | 1.078 | | 0.906 | 1.284 |
|  |  | Weighted median |  | 0.542 | 1.079 | | 0.844 | 1.380 |
|  |  | MR Egger |  | 0.309 | 1.314 | | 0.785 | 2.199 |
|  |  | Weighted mode |  | 0.648 | 1.080 | | 0.780 | 1.495 |
|  |  | Simple mode |  | 0.909 | 1.023 | | 0.696 | 1.505 |
| HT |  | IVW | 12 | 0.031 | 1.396 | | 1.030 | 1.892 |
|  |  | Weighted median |  | 0.084 | 1.437 | | 0.952 | 2.167 |
|  |  | MR Egger |  | 0.157 | 1.962 | | 0.827 | 4.655 |
|  |  | Weighted mode |  | 0.170 | 1.447 | | 0.883 | 2.370 |
|  |  | Simple mode |  | 0.330 | 1.385 | | 0.740 | 2.591 |
| Hypothyroidism |  | IVW | 65 | 0.002 | 1.431 | | 1.138 | 1.799 |
|  |  | Weighted median |  | 0.035 | 1.472 | | 1.028 | 2.108 |
|  |  | MR Egger |  | 0.006 | 1.990 | | 1.234 | 3.210 |
|  |  | Weighted mode |  | 0.071 | 1.443 | | 0.976 | 2.132 |
|  |  | Simple mode |  | 0.979 | 1.009 | | 0.521 | 1.952 |
| Hyperthyroidism |  | IVW | 11 | 0.974 | 0.996 | | 0.757 | 1.308 |
|  |  | Weighted median |  | 0.590 | 1.110 | | 0.758 | 1.626 |
|  |  | MR Egger |  | 0.274 | 0.679 | | 0.355 | 1.302 |
|  |  | Weighted mode |  | 0.603 | 1.161 | | 0.674 | 1.999 |
|  |  | Simple mode |  | 0.569 | 1.219 | | 0.631 | 2.357 |
| TC |  | IVW | 258 | 0.669 | 1.004 | | 0.987 | 1.020 |
|  |  | Weighted median |  | 0.362 | 1.012 | | 0.986 | 1.039 |
|  |  | MR Egger |  | 0.727 | 1.009 | | 0.960 | 1.060 |
|  |  | Weighted mode |  | 0.396 | 1.023 | | 0.971 | 1.079 |
|  |  | Simple mode |  | 0.811 | 1.010 | | 0.933 | 1.092 |
| TSH |  | IVW | 22 | 0.847 | 1.035 | | 0.730 | 1.466 |
|  |  | Weighted median |  | 0.875 | 1.038 | | 0.653 | 1.648 |
|  |  | MR Egger |  | 0.768 | 1.188 | | 0.383 | 3.687 |
|  |  | Weighted mode |  | 0.781 | 0.904 | | 0.447 | 1.828 |
|  |  | Simple mode |  | 0.370 | 0.660 | | 0.271 | 1.604 |
| TRH |  | IVW | 26 | 0.386 | 1.162 | | 0.827 | 1.633 |
|  |  | Weighted median |  | 0.861 | 0.963 | | 0.628 | 1.476 |
|  |  | MR Egger |  | 0.275 | 1.680 | | 0.676 | 4.175 |
|  |  | Weighted mode |  | 0.622 | 0.852 | | 0.455 | 1.597 |
|  |  | Simple mode |  | 0.680 | 0.864 | | 0.434 | 1.718 |
| THRɑ |  | IVW | 16 | 0.942 | 0.986 | | 0.678 | 1.435 |
|  |  | Weighted median |  | 0.374 | 0.788 | | 0.466 | 1.333 |
|  |  | MR Egger |  | 0.835 | 1.100 | | 0.457 | 2.645 |
|  |  | Weighted mode |  | 0.516 | 0.730 | | 0.289 | 1.845 |
|  |  | Simple mode |  | 0.522 | 0.724 | | 0.275 | 1.905 |
| TP |  | IVW | 20 | 0.828 | 0.968 | | 0.723 | 1.297 |
|  |  | Weighted median |  | 0.550 | 0.881 | | 0.580 | 1.337 |
|  |  | MR Egger |  | 0.892 | 0.954 | | 0.490 | 1.859 |
|  |  | Weighted mode |  | 0.707 | 0.905 | | 0.544 | 1.508 |
|  |  | Simple mode |  | 0.984 | 1.007 | | 0.514 | 1.971 |
| TG |  | IVW | 23 | 0.460 | 1.117 | | 0.834 | 1.496 |
|  |  | Weighted median |  | 0.708 | 1.083 | | 0.714 | 1.644 |
|  |  | MR Egger |  | 0.952 | 1.020 | | 0.542 | 1.919 |
|  |  | Weighted mode |  | 0.911 | 1.041 | | 0.516 | 2.102 |
|  |  | Simple mode |  | 0.186 | 1.681 | | 0.797 | 3.543 |
| TBG |  | IVW | ---- | ---- | ---- | | ---- | ---- |
|  |  | Weighted median |  |  |  |  |  |  |
|  |  | MR Egger |  |  |  |  |  |  |
|  |  | Weighted mode |  |  |  |  |  |  |
|  |  | Simple mode |  |  |  |  |  |  |
| AA | GD | IVW | 18 | 0.114 | 1.025 | | 0.994 | 1.057 |
|  |  | Weighted median |  | 0.305 | 1.022 | | 0.980 | 1.067 |
|  |  | MR Egger |  | 0.907 | 1.003 | | 0.949 | 1.061 |
|  |  | Weighted mode |  | 0.102 | 1.059 | | 0.992 | 1.130 |
|  |  | Simple mode |  | 0.118 | 1.065 | | 0.988 | 1.148 |
|  | HT | IVW | 18 | 0.141 | 1.013 | | 0.996 | 1.032 |
|  |  | Weighted median |  | 0.193 | 1.017 | | 0.992 | 1.042 |
|  |  | MR Egger |  | 0.146 | 1.023 | | 0.993 | 1.054 |
|  |  | Weighted mode |  | 0.521 | 1.015 | | 0.970 | 1.062 |
|  |  | Simple mode |  | 0.551 | 1.014 | | 0.969 | 1.062 |
|  | Hypothyroidism | IVW | 18 | 0.376 | 1.004 | | 0.995 | 1.014 |
|  |  | Weighted median |  | 0.825 | 1.002 | | 0.987 | 1.016 |
|  |  | MR Egger |  | 0.282 | 1.008 | | 0.994 | 1.023 |
|  |  | Weighted mode |  | 0.848 | 0.997 | | 0.970 | 1.026 |
|  |  | Simple mode |  | 0.996 | 1.000 | | 0.975 | 1.026 |
|  | Hyperthyroidism | IVW | 18 | 0.123 | 1.020 | | 0.995 | 1.046 |
|  |  | Weighted median |  | 0.455 | 1.013 | | 0.979 | 1.049 |
|  |  | MR Egger |  | 0.377 | 1.019 | | 0.979 | 1.061 |
|  |  | Weighted mode |  | 0.508 | 1.015 | | 0.972 | 1.059 |
|  |  | Simple mode |  | 0.867 | 1.005 | | 0.949 | 1.065 |
|  | TC | IVW | 5 | 0.985 | 0.998 | | 0.795 | 1.253 |
|  |  | Weighted median |  | 0.752 | 0.962 | | 0.755 | 1.226 |
|  |  | MR Egger |  | 0.561 | 1.739 | | 0.329 | 9.187 |
|  |  | Weighted mode |  | 0.764 | 0.946 | | 0.674 | 1.327 |
|  |  | Simple mode |  | 0.663 | 0.913 | | 0.626 | 1.333 |
|  | TSH | IVW | 17 | 0.470 | 0.009 | | -0.016 | 0.035 |
|  |  | Weighted median |  | 0.531 | 0.011 | | -0.024 | 0.046 |
|  |  | MR Egger |  | 0.338 | 0.019 | | -0.019 | 0.057 |
|  |  | Weighted mode |  | 0.385 | 0.018 | | -0.022 | 0.059 |
|  |  | Simple mode |  | 0.636 | 0.018 | | -0.054 | 0.089 |
|  | TRH | IVW | 17 | 0.009 | -0.029 | | -0.051 | -0.007 |
|  |  | Weighted median |  | 0.076 | -0.026 | | -0.056 | 0.003 |
|  |  | MR Egger |  | 0.092 | -0.029 | | -0.061 | 0.003 |
|  |  | Weighted mode |  | 0.140 | -0.026 | | -0.058 | 0.007 |
|  |  | Simple mode |  | 0.199 | -0.029 | | -0.073 | 0.014 |
|  | THRɑ | IVW | 17 | 0.625 | 0.005 | | -0.017 | 0.027 |
|  |  | Weighted median |  | 0.947 | 0.001 | | -0.030 | 0.032 |
|  |  | MR Egger |  | 0.322 | 0.017 | | -0.015 | 0.049 |
|  |  | Weighted mode |  | 0.354 | -0.022 | | -0.066 | 0.023 |
|  |  | Simple mode |  | 0.371 | -0.026 | | -0.083 | 0.030 |
|  | TP | IVW | 17 | 0.224 | 0.015 | | -0.009 | 0.039 |
|  |  | Weighted median |  | 0.082 | 0.029 | | -0.004 | 0.062 |
|  |  | MR Egger |  | 0.527 | 0.012 | | -0.024 | 0.048 |
|  |  | Weighted mode |  | 0.244 | 0.026 | | -0.016 | 0.067 |
|  |  | Simple mode |  | 0.174 | 0.039 | | -0.015 | 0.094 |
|  | TG | IVW | 17 | 0.924 | 0.001 | | -0.021 | 0.023 |
|  |  | Weighted median |  | 0.455 | -0.011 | | -0.041 | 0.018 |
|  |  | MR Egger |  | 0.450 | -0.013 | | -0.044 | 0.019 |
|  |  | Weighted mode |  | 0.458 | -0.012 | | -0.044 | 0.020 |
|  |  | Simple mode |  | 0.543 | -0.013 | | -0.056 | 0.029 |
|  | TBG | IVW | 4 | 0.140 | -0.050 | | -0.116 | 0.016 |
|  |  | Weighted median |  | 0.121 | -0.059 | | -0.133 | 0.016 |
|  |  | MR Egger |  | 0.350 | -0.066 | | -0.173 | 0.041 |
|  |  | Weighted mode |  | 0.202 | -0.058 | | -0.129 | 0.012 |
|  |  | Simple mode |  | 0.193 | -0.099 | | -0.216 | 0.017 |

MR, Mendelian randomization; AA, alopecia areata; TD, thyroid dysfunction; SNP, single-nucleotide polymorphism; OR, odds ratio; CI, confidence interval; GD, Graves' disease; HT, Hashimoto thyroiditis; TC, thyroid cancer; TSH, thyroid stimulating hormone; TRH, thyrotropin-releasing hormone, TBG, thyroxine-binding globulin; THRα, thyroid hormone receptor alpha; TP, thyroid peroxidase; TG, thyroglobulin.
