# Supplementary material for: The genetic link between thyroid dysfunction and alopecia areata: a bidirectional two-sample Mendelian randomization study
Source: Front Endocrinol (Lausanne). 2024 Aug 14;15:1440941. doi: 10.3389/fendo.2024.1440941 (PMC11349512; doi:10.3389/fendo.2024.1440941)
Supplement: Supplementary file 10 [file Table4.docx]

***Supplementary Table S4*:** Pleiotropy and heterogeneity test between TD and AA.

| **Exposure** | **Outcome** | **Heterogeneity** | | | **Pleiotropy test** | | | **MR-PRESSO** | | |
| --- | --- | --- | --- | --- | --- | --- | --- | --- | --- | --- |
|  |  | ***P*** | **Q** | **Q_df** | ***P*** | **SE** | **Intercept** | **outliers** | **Global Test** | |
|  |  |  |  |  |  |  |  |  | **RSSobs** | ***P*** |
| GD | AA | 0.591 | 22.77 | 25 | 0.431 | 0.056 | -0.045 | 0 | 24.38 | 0.604 |
| HT |  | 0.432 | 11.13 | 11 | 0.428 | 0.077 | -0.063 | 0 | 12.64 | 0.49 |
| Hypothyroidism |  | 0.339 | 64 | 68.13 | 0.129 | 0.026 | -0.039 | 0 | 69.86 | 0.354 |
| Hyperthyroidism |  | 0.600 | 8.294 | 10 | 0.236 | 0.074 | 0.094 | 0 | 11.18 | 0.526 |
| TC |  | 0.134 | 282.2 | 257 | 0.827 | 0.017 | -0.004 | 0 | 284.4 | 0.196 |
| TSH |  | 0.087 | 30.24 | 21 | 0.804 | 0.102 | -0.026 | 0 | 33.09 | 0.099 |
| TRH |  | 0.094 | 34.67 | 25 | 0.401 | 0.077 | -0.066 | 0 | 37.04 | 0.104 |
| THRɑ |  | 0.596 | 13.08 | 15 | 0.792 | 0.122 | -0.033 | 0 | 14.97 | 0.606 |
| TP |  | 0.649 | 16.13 | 19 | 0.963 | 0.065 | 0.003 | 0 | 17.46 | 0.692 |
| TG |  | 0.554 | 20.46 | 22 | 0.754 | 0.059 | 0.019 | 0 | 22.19 | 0.563 |
| TBG |  | ---- | ---- | ---- | ---- | ---- | ---- | ---- | ---- | ---- |
| AA | GD | 0.340 | 18.80 | 17 | 0.381 | 0.019 | 0.017 | 0 | 20.83 | 0.382 |
|  | HT | 0.795 | 12.09 | 17 | 0.435 | 0.012 | -0.009 | 0 | 13.78 | 0.781 |
|  | Hypothyroidism | 0.788 | 12.19 | 17 | 0.491 | 0.006 | -0.004 | 0 | 15.20 | 0.716 |
|  | Hyperthyroidism | 0.254 | 20.41 | 17 | 0.952 | 0.016 | 0.001 | 0 | 22.48 | 0.267 |
|  | TC | 0.184 | 6.21 | 4 | 0.556 | 0.415 | -0.275 | 0 | 10.62 | 0.205 |
|  | TSH | 0.151 | 21.76 | 16 | 0.501 | 0.018 | -0.012 | 0 | 24.79 | 0.155 |
|  | TRH | 0.963 | 7.47 | 16 | 0.998 | 0.015 | 0.000 | 0 | 8.02 | 0.969 |
|  | THRɑ | 0.737 | 12.10 | 16 | 0.358 | 0.015 | -0.014 | 0 | 14.45 | 0.69 |
|  | TP | 0.240 | 19.58 | 16 | 0.822 | 0.017 | 0.004 | 0 | 22.44 | 0.252 |
|  | TG | 0.576 | 14.30 | 16 | 0.263 | 0.015 | 0.018 | 0 | 15.65 | 0.618 |
|  | TBG | 0.317 | 3.53 | 3 | 0.713 | 0.049 | 0.021 | 0 | 4.34 | 0.591 |

AA, Alopecia areata; TD, thyroid dysfunction: SNP, single-nucleotide polymorphism; GD, Graves' disease; HT, Hashimoto thyroiditis; TC, thyroid cancer; TSH, thyroid stimulating hormone; TRH, thyrotropin-releasing hormone, TBG, thyroxine-binding globulin; THRα, thyroid hormone receptor alpha; TP, thyroid peroxidase; TG, thyroglobulin.
