# Supplementary material for: The genetic link between thyroid dysfunction and alopecia areata: a bidirectional two-sample Mendelian randomization study
Source: Front Endocrinol (Lausanne). 2024 Aug 14;15:1440941. doi: 10.3389/fendo.2024.1440941 (PMC11349512; doi:10.3389/fendo.2024.1440941)
Supplement: Supplementary file 11 [file Table5.docx]

***Supplementary Table S5*:** STROBE-MR checklist of recommended items to address in reports of Mendelian randomization studies.

**STROBE-MR checklist of recommended items to address in reports of Mendelian randomization studies**^1^ ^2^

| **Item No.** | **Section** | **Checklist item** | **Page No.** | **Relevant text from manuscript** |
| --- | --- | --- | --- | --- |
| 1 | **TITLE and ABSTRACT** | Indicate Mendelian randomization (MR) as the study’s design in the title and/or the abstract if that is a main purpose of the study |  | Title：The genetic link between Thyroid dysfunction and Alopecia areata: A bidirectional two-sample Mendelian randomization study  Abstract:  Introduction:  Although descriptive studies have found an association between thyroid dysfunction (TD) and alopecia areata (AA), however, the causal relationship between TD and AA remains unclear. Mendelian randomization (MR) was used to explore the causal relationship between TD and AA.  Methods:  We performed large-scale, two-sample Mendelian randomization (MR) analyses to examine whether there was an association between TD (such as Graves’ disease (GD), Hashimoto's thyroiditis (HT), thyroid cancer (TC), thyroid stimulating hormone (TSH), thyrotropin-releasing hormone (TRH), etc.) and AA. Genome-wide association study (GWAS) summary statistics for TD and AA were from the IEU Open Gwas project. The inverse variance-weighted (IVW) method was used as the primary analysis method to evaluate the causality between TD and AA, supplemented by the weighted median, MR-Egger, simple mode and weighted mode. In addition, sensitivity analyses were performed to assess the reliability of the study results.  Results:  Our study found that single nucleotide polymorphisms (SNPs) in HT (IVW OR = 1.396, 95% CI 1.030-1.892, P=0.031) and hypothyroidism (IVW OR = 1.431, 95% CI 1.138-1.799, P=0.002) significantly increased the risk of AA. Reverse MR analysis indicated that genetic susceptibility to AA (β=-0.029, 95%CI=-0.051 to -0.007, P=0.009) may be a risk for TRH. Positive MR analysis observed no statistically significant causal relationship between other TD and AA (IVW P>0.05). Reverse MR analysis also showed no statistically significant association between AA and other TD (IVW P>0.05) other than TRH. Furthermore, additional sensitivity analyses were performed, including a leave-one-out test, a heterogeneity test, and a pleiotropy test to assess the robustness of the results.  Conclusion:  This study provides a very comprehensive analysis of the causal relationship between TD and AA, providing convincing genetic evidence to support the causal relationship between TD and alopecia areata. It reveals some causes of AA patients, which is of great significance for the management and treatment of AA patients. |
|  | **INTRODUCTION** |  |  |  |
| 2 | **Background** | Explain the scientific background and rationale for the reported study. What is the exposure? Is a potential causal relationship between exposure and outcome plausible? Justify why MR is a helpful method to address the study question |  | Scientific Background and Rationale:  Alopecia areata (AA) is a common autoimmune disease characterized by non-scarring alopecia. Most AA patients experience unpredictable relapse and remission. The disease not only reduces the quality of life of patients, but also may lead to depression, anxiety and other emotional disorders. A meta-analysis of multiple observational studies showed that people with TD had an increased risk of AA than those without TD; however, the causal relationship between TD and AA is unclear.  Exposure and Potential Causal Relationship:  Exposure: GD, HT, hyperthyroidism, hypothyroidism, TC, TSH, TRH, TBG, THRα, TP and TG.  Causal Relationship Plausibility: The causal relationship between TD and AA is credible, and a large number of observational studies have shown that there is a correlation between TD and AA. AA shares an autoimmune background with autoimmune thyroid disease, either sporadic or autoimmune polyglandular syndrome. Thyroid hormones are essential for the growth and maintenance of hair follicles. In patients with hypothyroidism, the epidermis is thinner and hair loss occurs frequently, indicating that thyroid hormone signals can regulate skin proliferation and hair growth.  Justification for Using MR:  Addressing Confounding and Reverse Causation: Traditional observational studies exploring the relationship between TD and AA may be affected by confounding factors and reverse causation. The MR method, using genetic variants as instrumental variables, helps overcome these issues, providing more reliable causal inference.  Genetic Instrumentation: In this study, SNPs directly associated with TD were chosen as instrumental variables, which are less likely to be influenced by confounding factors related to AA risk.  Robustness to Confounding: A major advantage of the MR method is its robustness against traditional confounding factors, making it a powerful tool for exploring the relationship between TD and AA. |
| 3 | **Objectives** | State specific objectives clearly, including pre-specified causal hypotheses (if any). State that MR is a method that, under specific assumptions, intends to estimate causal effects |  | The aim of this study was to investigate the causal association between TD (GD, HT, hyperthyroidism, hypothyroidism, TC, TSH, TRH, TBG, THRα, TP and TG) and AA through Mendelian randomization (MR) analysis. |
|  | **METHODS** |  |  |  |
| 4 | **Study design and data sources** | Present key elements of the study design early in the article. Consider including a table listing sources of data for all phases of the study. For each data source contributing to the analysis, describe the following: |  |  |
|  | a) | Setting: Describe the study design and the underlying population, if possible. Describe the setting, locations, and relevant dates, including periods of recruitment, exposure, follow-up, and data collection, when available. |  | Design Overview: This study employs a two-sample Mendelian Randomization approach to explore the causal relationship between TD and AA.  Population Basis: The analysis utilizes genetic data from large-scale genome-wide association studies (GWAS), representing a broad European population.  Data Sources:  GWAS Data for TD: GWAS summary data for TD, including GD, HT, hyperthyroidism, hypothyroidism, TC, TSH, TRH, TBG, THRα, TP and TG, were obtained from the IEU-Open GWAS. See Supplementary Table S1 for details.  GWAS Data for AA: Original data for alopecia areata came from the FinnGen study (accessed through <https://www.finngen.fi/en/access_results>). See Supplementary Table S1 for details.  Setting and Locations:  Data Origin: The GWAS data used in this study are derived from large-scale studies conducted in European populations.  Study Scope: While based on European data, the findings have broader implications for understanding the role of TD in AA globally.  Relevant Dates:  Data Collection Period: The original TD GWAS data were collected in 2013-2021, and the original AA GWAS data were collected in 2021.  Recruitment, Exposure, Follow-up:  Recruitment and Exposure: As this study is a secondary analysis of existing GWAS data, details of recruitment and exposure pertain to the original GWAS studies.  Follow-up and Data Collection: Follow-up and data collection periods are as per the original GWAS datasets, with the focus of this MR study being on the analysis of pre-existing data. |
|  | b) | Participants: Give the eligibility criteria, and the sources and methods of selection of participants. Report the sample size, and whether any power or sample size calculations were carried out prior to the main analysis |  | Eligibility Criteria and Participant Selection:  Criteria: The study utilized pre-existing GWAS datasets, hence the eligibility criteria and participant selection were as per the original GWAS studies. These studies typically include criteria based on age, health status, and specific genetic markers.  Sample Size:  GWAS Data for TD and AA: Informations on population and instrumental variables for TD and AA GWAS data were provided in Supplementary Table S1. |
|  | c) | Describe measurement, quality control and selection of genetic variants |  | Measurement of Genetic Variants:  Data Source: The measurement of genetic variants was conducted as part of the GWAS datasets. Specific methods of genetic measurement, including SNP genotyping platforms, are detailed in the original GWAS publications.  Quality Control:  Data Filtering: The GWAS data underwent rigorous quality control measures, including filtering for call rate, minor allele frequency, and Hardy-Weinberg equilibrium, as detailed in the original GWAS methodology.  Selection of Genetic Variants:  Criteria for Instrumental Variables: In this MR study, SNPs were selected as instrumental variables based on their association with TD. The selection criteria included a P-value of less than 5×10⁻^8^/1×10⁻^5^ from the GWAS data related to TD. SNPs exhibiting linkage disequilibrium (LD) r² values greater than 0.001 within a 10000 kb range were filtered out using the PLINK tool.  Instrument Strength: The strength of the selected SNPs as instrumental variables was assessed by calculating the F-statistic for each immune trait, ensuring they are strong and valid instruments for the MR analysis. |
|  | d) | For each exposure, outcome, and other relevant variables, describe methods of assessment and diagnostic criteria for diseases |  | Exposure Assessment:  TD Phenotypes (GD, HT, hyperthyroidism, hypothyroidism, TC, TSH, TRH, TBG, THRα, TP and TG): These were assessed using IEU online open GWAS statistics, including ebi-a-GCST90018847, ebi-a-GCST90018855, ebi-a-GCST90018862, ebi-a-GCST90018860, ieu-a-1082, prot-a-530, prot-a-3102, prot-a-2974, prot-a-3088, prot-a-2960, prot-c-2706_69_2. The diagnosis of GD, HT, hyperthyroidism, hypothyroidism and TC are based on established clinical and histological parameters. TSH, TRH, TBG, THRα, TP and TG are indicators of thyroid function.  Outcome Assessment:  AA Subtypes: Original data for alopecia areata came from the FinnGen study,the diagnosis of AA was determined according to the International Classification of Diseases 9th Revision (ICD-8, ICD-9, ICD-10）codes. The GWAS data of AA in this study was from the IEU open GWAS project (ID: finn-b-L12 _ ALOPECAREATA).  Other Relevant Variables:  Confounding Factors: Potential confounders, such as genetic factors, mental stress (Such as anxiety, depression, insomnia, etc.) and intestinal dysbiosis were assessed using the LDlink website ((https://ldlink.nih.gov/?tab=ldtrait)). This assessment helped ensure that the selected SNPs for MR analysis were independent of these established risk factors. |
|  | e) | Provide details of ethics committee approval and participant informed consent, if relevant |  | Ethics Committee Approval:  Approval Details: This study is based on the analysis of pre-existing GWAS datasets. The original studies from which these data were derived obtained ethics committee approval. Details of these approvals can be found in the original GWAS publications.  Participant Informed Consent:  Consent Process: The participants in the original GWAS studies provided informed consent for their genetic data to be used in research. The process and documentation of informed consent are described in the methodology section of the original GWAS studies. |
| 5 | **Assumptions** | Explicitly state the three core IV assumptions for the main analysis (relevance, independence and exclusion restriction) as well assumptions for any additional or sensitivity analysis |  | Core IV Assumptions for Main Analysis:  Relevance: Genetic variants (IVs) are strongly associated with TD.  Independence: IVs are independent of confounders affecting both exposure and outcome.  Exclusion Restriction: IVs influence the outcome only through the exposure.  Assumptions for Additional or Sensitivity Analysis:  Weighted Median Method: Assumes that at least 50% of the information comes from valid instrumental variables (IVs).  MR-Egger: Assumes balanced pleiotropic effects.  MR-PRESSO: Used for detecting and correcting outliers in instrumental variable analysis.  F-Statistics: Employed to calculate statistical power, ensuring the strength and validity of the selected genetic variants as instrumental variables.  Q-Statistic: Used to detect heterogeneity among the instrumental variables.  False Discovery Rate (FDR): Applied in multiple testing to correct for the probability of false positive findings. |
| 6 | **Statistical methods: main analysis** | Describe statistical methods and statistics used |  |  |
|  | a) | Describe how quantitative variables were handled in the analyses (i.e., scale, units, model) |  | Handling of Quantitative Variables:  Scale and Units: The quantitative variables, primarily TD phenotypes, were analyzed using their respective scales and units as reported in the GWAS datasets.  Statistical Model: The study employed the inverse variance weighted (IVW) method as the primary model for Mendelian Randomization analysis. This method combines the Wald estimates for each SNP to estimate the overall effect of the exposure on the outcome.  Data Transformation: No data transformation was performed for the analysis. |
|  | b) | Describe how genetic variants were handled in the analyses and, if applicable, how their weights were selected |  | Handling of Genetic Variants:  Variant Selection: In this study, SNPs were selected as instrumental variables based on a P-value of less than 1×10^⁻5^/5×10^⁻8^ from the GWAS data related to each TD trait. SNPs exhibiting linkage disequilibrium (LD) r² values greater than 0.001 within a 10000 kb range were filtered out using the PLINK tool.  Weight Selection: For the inverse variance weighted (IVW) method, the weights of each SNP were determined by the precision of their estimates, typically using the inverse of the variance of the Wald ratio for each SNP. |
|  | c) | Describe the MR estimator (e.g. two-stage least squares, Wald ratio) and related statistics. Detail the included covariates and, in case of two-sample MR, whether the same covariate set was used for adjustment in the two samples |  | MR Estimator and Statistics:  MR Estimator: The primary MR estimator used in this study was the inverse variance weighted (IVW) method. This approach combines the Wald ratios of individual SNPs to estimate the overall causal effect.  Related Statistics: Additional statistical methods for sensitivity analysis, such as MR-Egger regression and the weighted median estimator, were likely employed to assess the robustness of the results and to test for potential pleiotropic effects.  Covariates:  No adjustment was made for covariates |
|  | d) | Explain how missing data were addressed |  | Not applicable. |
|  | e) | If applicable, indicate how multiple testing was addressed |  | Addressing Multiple Testing:  The study applied False Discovery Rate (FDR) correction to address the issue of multiple testing, particularly relevant when analyzing a large number of genetic variants. This method adjusts the p-values to reduce the risk of type I errors (false positives) in the context of multiple comparisons. |
| 7 | **Assessment of assumptions** | Describe any methods or prior knowledge used to assess the assumptions or justify their validity |  | The study employed F-statistics and MR-Egger intercept to assess the validity of the core assumptions of Mendelian Randomization. |
| 8 | **Sensitivity analyses and additional analyses** | Describe any sensitivity analyses or additional analyses performed (e.g. comparison of effect estimates from different approaches, independent replication, bias analytic techniques, validation of instruments, simulations) |  | Initially, the Q-test method was utilized to evaluate potential heterogeneity among individual IVs, and p-value less than 0.05 from the Cochran Q test is considered indicative of heterogeneity in the results. Subsequently, the MR‐Egger intercept test was applied to estimate horizontal pleiotropy, guaranteeing that genetic variation has an independent relationship with both the exposure and outcome. We used MR-PRESSO to re-examine the presence of heterogeneous SNPs. Additionally, we conducted a leave-one-out (LOO) analysis, assessing whether the results were significantly influenced by individual SNPs by sequentially dropping each SNP and then performing MR analysis.  Reverse MR analysis: We regard AA as exposure and TD as result. |
| 9 | **Software and pre-registration** |  |  |  |
|  | a) | Name statistical software and package(s), including version and settings used |  | All statistical analyses were performed using R version 4.3.0. Specifically, for MR analysis, we employed the TwoSampleMR0.5.9, forestploter4.3.2. |
|  | b) | State whether the study protocol and details were pre-registered (as well as when and where) |  | The study did not involve pre-registration of the study protocol. |
|  | **RESULTS** |  |  |  |
| 10 | **Descriptive data** |  |  |  |
|  | a) | Report the numbers of individuals at each stage of included studies and reasons for exclusion. Consider use of a flow diagram |  | Demographic data, such as the number of people in each dataset included in the study, are shown in Supplementary Table S1. |
|  | b) | Report summary statistics for phenotypic exposure(s), outcome(s), and other relevant variables (e.g. means, SDs, proportions) |  | Relevant summary statistics were not available |
|  | c) | If the data sources include meta-analyses of previous studies, provide the assessments of heterogeneity across these studies |  | Data sources did not include meta-analyses of previous studies |
|  | d) | For two-sample MR:  i.  Provide justification of the similarity of the genetic variant-exposure associations between the exposure and outcome samples  ii.  Provide information on the number of individuals who overlap between the exposure and outcome studies |  | i.  All the sample populations listed in Supplementary Table S1 were from Europe and thus showed little ethnic heterogeneity  ii. We utilized GWAS datasets from various databases for exposure and outcome to mitigate potential bias in causal effect estimation due to sample overlap. |
| 11 | **Main results** |  |  |  |
|  | a) | Report the associations between genetic variant and exposure, and between genetic variant and outcome, preferably on an interpretable scale |  | Relationships among exposures, outcomes, and covariates were interpreted in directed acyclic graphs (Figure 1). |
|  | b) | Report MR estimates of the relationship between exposure and outcome, and the measures of uncertainty from the MR analysis, on an interpretable scale, such as odds ratio or relative risk per SD difference |  | Our study found that single nucleotide polymorphisms (SNPs) in HT (IVW OR = 1.396, 95% CI 1.030-1.892, P=0.031) and hypothyroidism (IVW OR = 1.431, 95% CI 1.138-1.799, P=0.002) significantly increased the risk of AA. Reverse MR analysis indicated that genetic susceptibility to AA (β=-0.029, 95%CI=-0.051 to -0.007, P=0.009) may be a risk for TRH. Positive MR analysis observed no statistically significant causal relationship between other TD and AA (IVW P>0.05). Reverse MR analysis also showed no statistically significant association between AA and other TD (IVW P>0.05) other than TRH.  The specific results are shown in Figure 2-5. |
|  | c) | If relevant, consider translating estimates of relative risk into absolute risk for a meaningful time period |  | No report |
|  | d) | Consider plots to visualize results (e.g. forest plot, scatterplot of associations between genetic variants and outcome versus between genetic variants and exposure) |  | The study includes various plots to visualize the results of the MR analysis. These may include forest plots for displaying MR estimates for TD subtypes associated with AA subtypes, and scatterplots to illustrate the associations between genetic variants and AA subtypes versus the associations between genetic variants and TD. |
| 12 | **Assessment of assumptions** |  |  |  |
|  | a) | Report the assessment of the validity of the assumptions |  | The example text reports the results of the assessment of the validity of relevant assumptions in multiple places throughout the main text and the appendices, such as in Supplementary Tables S2, S4. It reports the statistical power of the instrumental variables in each association, represented by F-statistic values. The Q-statistic is used to detect heterogeneity in the statistical model, assessing its stability. |
|  | b) | Report any additional statistics (e.g., assessments of heterogeneity across genetic variants, such as *I^2^*, Q statistic or E-value) |  | The study includes assessments of heterogeneity across the genetic variants used as instrumental variables, utilizing the Q statistic (Q test). Such as in Supplementary Table S4. |
| 13 | **Sensitivity analyses and additional analyses** |  |  |  |
|  | a) | Report any sensitivity analyses to assess the robustness of the main results to violations of the assumptions |  | We used the MR-PRESSO method to detect and remove abnormal SNPs, used the LDlink website to exclude confounding factors related SNPs, and then performed MR analysis again. To assess the robustness of the results, a series of sensitivity analyses were further performed, including the use of mr_pleiotropy test and the intercept term in MR-Egger to check for the presence of horizontal pleiotropy, and the use of mr_heterogeneity to detect the presence of heterogeneity. At the same time, a scatter plot of effective IV was used to estimate the causal effect of TD on AA to visually assess whether the outcome effect was zero when the IV effect was zero. In addition, the leave-one-out method was used to cull each SNP one by one, and then the meta-effect of the remaining SNPs was calculated to see if the results changed significantly after the removal of specific SNPs. For details, see Figure 3-5, Supplementary Figure S1-S5. |
|  | b) | Report results from other sensitivity analyses or additional analyses |  | No report |
|  | c) | Report any assessment of direction of causal relationship (e.g., bidirectional MR) |  | We conducted a reverse causal analysis, treating AA as an exposure and TD as a result.Reverse MR analysis indicated that genetic susceptibility to AA (β=-0.029, 95%CI=-0.051 to -0.007, P=0.009) may be a risk for TRH. Reverse MR analysis also showed no statistically significant association between AA and other TD (IVW P>0.05) other than TRH. |
|  | d) | When relevant, report and compare with estimates from non-MR analyses |  | No report |
|  | e) | Consider additional plots to visualize results (e.g., leave-one-out analyses) |  | For details, see Figure 3-5, Supplementary Figure S1-S5. |
|  | **DISCUSSION** |  |  |  |
| 14 | **Key results** | Summarize key results with reference to study objectives |  | Our study found that single nucleotide polymorphisms (SNPs) in HT (IVW OR = 1.396, 95% CI 1.030-1.892, P=0.031) and hypothyroidism (IVW OR = 1.431, 95% CI 1.138-1.799, P=0.002) significantly increased the risk of AA. Reverse MR analysis indicated that genetic susceptibility to AA (β=-0.029, 95%CI=-0.051 to -0.007, P=0.009) may be a risk for TRH. Positive MR analysis observed no statistically significant causal relationship between other TD and AA (IVW P>0.05). Reverse MR analysis also showed no statistically significant association between AA and other TD (IVW P>0.05) other than TRH. |
| 15 | **Limitations** | Discuss limitations of the study, taking into account the validity of the IV assumptions, other sources of potential bias, and imprecision. Discuss both direction and magnitude of any potential bias and any efforts to address them |  | Our studies also have some limitations. Firstly, due to the limited data available for GWAS in alopecia areata, this study failed to perform a confirmatory analysis. Secondly, our research population was all European, and the conclusions of the study may not be applicable to other ethnic groups. Finally, although our study provides genetic evidence for causality, additional research is needed to further elucidate the underlying mechanisms. |
| 16 | **Interpretation** |  |  |  |
|  | a) | Meaning: Give a cautious overall interpretation of results in the context of their limitations and in comparison with other studies |  | The example text dedicates the largest section to discussing this item, providing a reasonable interpretation of the MR results by comparing them with multiple published studies. |
|  | b) | Mechanism: Discuss underlying biological mechanisms that could drive a potential causal relationship between the investigated exposure and the outcome, and whether the gene-environment equivalence assumption is reasonable. Use causal language carefully, clarifying that IV estimates may provide causal effects only under certain assumptions |  | The article has explored the possible biological mechanisms. |
|  | c) | Clinical relevance: Discuss whether the results have clinical or public policy relevance, and to what extent they inform effect sizes of possible interventions |  | In summary, we conducted a two-way two-sample Mendelian randomization study to explore the causal relationship between TD and AA. Our study found that HT and hypothyroidism AA have causal effects, suggesting that HT and hypothyroidism itself may play a causal role in the pathogenesis of AA. There was no causal relationship between GD, hyperthyroidism, TC, TSH, TRH, TBG, THRα, TP, TG and AA. In addition, reverse MR analysis showed that the genetic susceptibility of AA may affect the risk of TRH. A series of sensitivity analyses supported these findings. Our results provide new insights into the occurrence of AA in TD patients, provide guidance for the treatment of AA patients, and help to improve the quality of life of AA patients. Continuous monitoring of thyroid-related hormones, timely diagnosis and treatment of TD can provide valuable insights into the management and lifestyle intervention of AA. |
| 17 | **Generalizability** | Discuss the generalizability of the study results (a) to other populations, (b) across other exposure periods/timings, and (c) across other levels of exposure |  | The text reports in the discussion that "Secondly, our research population was all European, and the conclusions of the study may not be applicable to other ethnic groups". |
|  | **OTHER INFORMATION** |  |  |  |
| 18 | **Funding** | Describe sources of funding and the role of funders in the present study and, if applicable, sources of funding for the databases and original study or studies on which the present study is based |  | We did not receive any funding for this study. |
| 19 | **Data and data sharing** | Provide the data used to perform all analyses or report where and how the data can be accessed, and reference these sources in the article. Provide the statistical code needed to reproduce the results in the article, or report whether the code is publicly accessible and if so, where |  | All the data used in this study are public. These analyses do not require the consent of human subjects. All data can be found in the IEU open GWAS project (https://gwas.mrcieu.ac.uk/datasets/). The catalog of all exposure and outcome-related GWAS datasets is shown in Supplementary Table S1. |
| 20 | **Conflicts of Interest** | All authors should declare all potential conflicts of interest |  | The authors declare that they have no known competing financial interests or personal relationships that could have appeared to influence the work reported in this paper. |

This checklist is copyrighted by the Equator Network under the Creative Commons Attribution 3.0 Unported (CC BY 3.0) license.

1. Skrivankova VW, Richmond RC, Woolf BAR, Yarmolinsky J, Davies NM, Swanson SA, et al. Strengthening the Reporting of Observational Studies in Epidemiology using Mendelian Randomization (STROBE-MR) Statement. JAMA. 2021;under review.

2. Skrivankova VW, Richmond RC, Woolf BAR, Davies NM, Swanson SA, VanderWeele TJ, et al. Strengthening the Reporting of Observational Studies in Epidemiology using Mendelian Randomisation (STROBE-MR): Explanation and Elaboration. BMJ. 2021;375:n2233.
